# The components and effects of home rehabilitation on activities of daily living and physical performance of community dwelling older people with low physical performance – a systematic review and meta-analysis of randomized controlled trials

**DOI:** 10.1186/s12877-026-07887-9

**Published:** 2026-06-30

**Authors:** Karin Högstedt, Wilhelmus Johannes Andreas Grooten, Maria Flink, Katarina Baudin, Susanne Guidetti, Elisabeth Rydwik

**Affiliations:** 1 Division of Occupational Therapy, Department of Neurobiology, Care Science & Society, Karolinska Institutet, Huddinge, Sweden; 2 Division of Physiotherapy, Department of Neurobiology, Care Science & Society, Karolinska Institutet, Huddinge, Sweden; 3 School of Health and Welfare, Dalarna University, Falun, Sweden; 4 Division of Family Medicine and Primary Care, Department of Neurobiology, Care Science & Society, Karolinska Institutet, Huddinge, Sweden; 5 Research and Development Unit for Older Persons, FOU nu, Stockholm, Sweden; 6 Theme Women’s Health and Allied Health Professionals, Medical Unit Allied Health Professionals, Karolinska University Hospital, Stockholm, Sweden

**Keywords:** Aged, Home-based rehabilitation, Independent living, Occupational therapy, Physiotherapy, Randomized controlled trials as topic

## Abstract

**Background:**

For people in old age, the risk of limitations in activities of daily living (ADL), low physical performance, and chronic diseases increases. Home rehabilitation targeting physical performance is a common intervention for older people with multimorbidity. Still research on home rehabilitation is mainly diagnosis specific. The objectives of this study were to summarize intervention components and evaluate the effects of home rehabilitation on ADL and physical performance in community-dwelling older people (65 years or older) with low physical performance and/or ADL difficulties.

**Methods:**

The databases MEDLINE, Web of Science, and CINAHL (January 2006–September 2025), plus references were screened, using keywords related to aging, home rehabilitation, ADL, and physical performance in randomized controlled trials (RCTs).

Inclusion criteria: RCTs of supervised home rehabilitation, targeting physical performance and/or basic and/or instrumental ADL (BADL/IADL) in community-dwelling people 65 years of age or older with low physical performance and/or ADL difficulties.

Exclusion criteria: scope of specific diagnoses, assisted living settings, centre-based, interventions areas outside the occupational therapy or physiotherapy disciplines, mainly delivered by home help service staff, exclusively outcomes outside the scope of ADL and physical performance conducted at home or non-English publications. 3,360 records were screened independently by two reviewers.

Data extraction followed the PROSPERO protocol. Methodological quality was assessed using the Joanna Briggs Institute RCT checklist (2020) (JBI), and certainty of evidence using Grading of Recommendations Assessment, Development, and Evaluation (GRADE). Random effects models generated pooled effects.

**Results:**

The review included 27 RCTs (*n* = 4,948), which were grouped into three intervention approaches. Twenty-three studies were graded as having low risk of bias, three as moderate risk of bias, and one of high risk of bias, using the JBI tool. The age of the participants ranged from 74 to 87 years of age.

Activity-based interventions improved BADL (*n* = 1,048, SMD 0.29, 95% CI 0.17 to 0.41, *P*<.001; moderate evidence) but not IADL (*n* = 603, SMD − 0.15, 95% CI − 0.31 to 0.01) or selected ADL tasks (*n* = 158, non-significant).

Exercise-based interventions improved BADL and physical performance (*n* = 310, SMD 0.43, 95% CI 0.21 to 0.66, *P*<.001; *n* = 1,472, SMD 0.20, 95% CI 0.10 to 0.30, *P*<.001), with low evidence due to imprecision and risk of bias according to GRADE.

Reablement-based interventions showed no significant effects on selected ADL tasks measured with Canadian Occupational Performance Measure (COPM) (*n* = 291, COPM performance MD 0.30, 95% CI − 0.25 to 0.86; COPM satisfaction MD 0.19, 95% CI − 0.04 to 0.42) or physical performance (*n* = 555, SMD 0.12, 95% CI − 0.05 to 0.28), with low to very low evidence according to GRADE.

**Conclusion:**

Home rehabilitation comprises three main intervention approaches. The Activity-based and Exercise-based yield small improvements in BADL and physical performance. Evidence for other ADL outcomes and the Reablement-based remains limited.

**Trial registration:**

PROSPERO (CRD42023488726).

**Supplementary Information:**

The online version contains supplementary material available at 10.1186/s12877-026-07887-9.

## Introduction

In older age, low physical performance is common [1] and the risk of multiple chronic diseases increases with negative impacts on functional abilities and quality of life [2]. For older people’s well-being and participation in daily life it is important to maintain functional abilities that enable a person to be active and mobile [3, 4]. For those with multimorbidity and low physical performance, evidence-based rehabilitation is a key factor addressing the older person’s abilities and environmental factors to optimize physical functioning [5].

Rehabilitation at home is common [6], for example after hospital discharge, as it utilizes the familiar environment and meaningful activities of daily living (ADL) to motivate training and enhance quality of life [7]. Home rehabilitation is mainly performed by physiotherapists (PTs) and occupational therapists (OTs) [8], targeting physical performance and ability in ADL [9]. Rehabilitation can also include reablement services which offer a multidisciplinary person-centered short-term intense home intervention period, grounded in a holistic approach to enhance a person’s functioning to pursue meaningful ADL [10]. Reablement services could differ between countries, as team composition varies, encompassing different combinations of healthcare professionals and, in some cases, social care staff. A key component, however, is that therapists instruct formal care workers to support older people receiving home-care services in becoming more independent [11].

Hence, home rehabilitation is often multidisciplinary and multicomponent, including components such as functional and person-centered goal setting, physical exercise and ADL training, self-care education, environmental modifications, provision of assistive products, and follow-up [9].

Multidisciplinary collaboration and coordination are described as essential components for adequate intervention delivery [5]. At the same time, reported challenges relating to collaboration and coordination in home rehabilitation include the need for clear communication to ensure client-centered goals, accurate self-training, maintaining motivation, and ensuring a smooth transition between health services [12]. Structured communication and trust [13] among professionals are important elements in joint work procedures for a holistic assessment of patient situation and personalized care plan to increase independence [5, 13].

Previous research indicates that supervision may be an effective component in rehabilitation [14]. Conducting rehabilitation within the home setting enables therapists to learn what works and what the patient needs to accomplish. Supervision during home visits facilitates interaction, reduce stress, convey feelings of safety and mastery [15], and individualize training progression. Nonetheless, patients’ consolidation of new skills may require further support over time [16]. Factors such as pain, fatigue, and unsteadiness can hinder adherence to training, demanding an extended intervention period, whereas developing strategies for self-training and self-care can support feeling in control [15].

Different multicomponent home rehabilitation interventions, delivered by OTs and PTs has been associated with positive outcomes in older people [14, 16–18]. OT approaches that combine intervention components of meaningful activities, client-centeredness, and empowerment have been shown to improve ADL and physical performance, and social participation in physically frail older adults [17]. Similarly, tailored multicomponent home-based exercise programs have been shown to be safe [18], and, when delivered by health professionals like PTs, enhance physical performance [18], and ADL after hospital discharge [14]. Additionally, ADL training, combining behavioral, environmental, and cognitive strategies has been found to increase performance among older people with various health conditions [16].

Rehabilitation outcomes may be impeded by several barriers. OT and PT have reported that limited time resources restrict their ability to tailor interventions according to the patient’s health conditions and support needs [19]. Environmental modifications appear beneficial for enhancing function and reducing fall risk, whereas other aspects, such as pain management and social participation, remain less studied [20]. The provision of assistive products also varies, depending on access to the professions [21].

Given the complexity of multicomponent home rehabilitation and its previous focus on diagnosis specific intervention, there is a lack of knowledge regarding the effect of home rehabilitation for people with low physical performance and several diseases. Clear descriptions of intervention components, their content and context, and effects, are necessary to support implementation and clinical use [22]. To strengthen the evidence-base of how home rehabilitation should be conducted, research identifying key components of the intervention, while acknowledging its clinical context, is needed. Although previous research has explored factors relevant to home rehabilitation, studies often focus on specific diagnoses [9, 23]. A broader analysis of randomized controlled trials (RCTs), investigating the effects on older people with low physical performance and ADL limitations, regardless of diagnosis or situation, may provide more generalizable evidence for clinical practice.

The aims of the study were (1) to summarize the components of home rehabilitation interventions used in RCTs on community-dwelling older (65 year or older) people with low physical performance and/or ADL difficulties, and (2) to evaluate the evidence on the effects of home rehabilitation on ADL and physical performance in this population. We hypothesized that home rehabilitation would improve ADL and physical performance.

## Methods

This systematic review and meta-analysis were registered in PROSPERO (CRD42023488726) and follows the Preferred Reporting Items for Systematic reviews and Meta-Analyses (PRISMA) checklist [24] (Additional file 1) addressing peer-reviewed RCTs, to reach a high level of scientific evidence [25].

### Eligibility criteria

Covering aspects of “PICO” (population, intervention, control, and outcomes), the inclusion criteria were: (P) Community-dwelling participants, mean age 65 or older, with low physical performance and/or difficulty performing at least one basic ADL (BADL) and/or instrumental ADL (IADL), (I) supervised rehabilitation intervention at the participant’s home, (C) compared to “usual care” as a control group, or “non-active interventions” or “no interventions” in RCTs, (O) effects on physical performance and/or BADL and/or IADL. The last two control group types were deviations from the PROSPERO protocol.

Exclusion criteria were: (1) RCTs in which the primary focus was on individuals with specific diagnoses or conditions, (2) People living in assisted care facilities, (3) Centre-based interventions, (4) Interventions not predominantly authorized or prescribed by OT or PT or in the area of the disciplines, or mainly delivered by home help service staff, (5) exclusively outcomes outside the scope of ADL and physical performance, and (6) articles not written in English.

### Data sources and searches

The systematic review is conducted within the multisite research program called Re@home in Sweden. The search for this study included RCTs, published during a period of 20 years, from January 1, 2006, to September 8, 2025, available in three scientific databases (MEDLINE, Web of Science, and CINAHL), covering a large area of relevant literature within rehabilitation, nursing, and medicine. The search strategy (Additional file 2) was developed in Medline (Ovid) in collaboration with librarians at the Karolinska Institutet University Library. For each search concept Medical Subject Headings (MeSH-terms) and free text terms were identified. Relevant studies [26–37] that evaluated the effects of home rehabilitation were identified, and these studies were used when validating the search strategy. The search was then translated into the other databases. Reference lists and systematic reviews were manually screened for further publications.

### Study selection and data extraction

Before the screening process, duplicates were removed by the University Library at the Karolinska Institutet. Thereafter, titles and abstracts were screened, and full-text articles were read independently by pairs of researchers (ER, KH) using the web-based software platform Covidence systematic review. To solve disagreements, a third member was consulted to find a consensus (SG).

Key study characteristics of relevance for the research questions (purpose of the study, population, intervention components, control components, outcome results), were selected and extracted by one reviewer (KH), in consultation with colleagues (ER, KB, SG). For example, assessment measurements were discussed as to how these answered the research questions, as studies used a variety of measurements. To structure characteristics of the interventions, the TIDieR template for RCTs [38] informed the description of providers, purpose, components, dosage, and adherence. The studies used different therapeutic approaches, which were grouped according to intervention purpose and components. E.g., activity-oriented or individually adjusted exercise program or reablement training program. This was done in order to group the home rehabilitation approaches for the meta-analysis. Extracted data are presented in tables.

Outcome results of BADL, IADL, combined BADL and IADL (reported as ADL), selected ADL tasks, and physical performance measurement tools were extracted for all published time points. If data for synthesis were missing, the authors of the selected studies were contacted. Retrieved data from authors are not shown [32]. Also, reported adverse events were summarized, a deviation from our PROSPERO protocol. BADL refers to fundamental self-care activities (e.g. bathing, dressing), whereas IADL involves more complex tasks necessary for independent living (e.g. shopping, meal preparation).

### Risk of bias assessment

To critically evaluate the methodological quality of the included studies, the Joanna Briggs Institute RCT checklist 2020 (JBI) [39] was used. The studies were assessed independently by two reviewers (ER, KH), and a third researcher was involved in disagreement discussions (SG). All through the assessments, the process was validated through discussions, comparing reports of the selected studies, and contacting the JBI administration for clarification of the tool. Three clarifying adjustments were made regarding the quality assessment tool: (1) baseline characteristics needed to differ by more than 10% between groups to qualify as a risk of bias (RoB); (2) impact analysis would include reasons for loss to follow-up per group; (3) other impact analyses were not required.

The JBI does not provide a RoB cut-off. However, cut-offs in a recent RCT review [40], that only used 12 of the possible 13 assessment questions, excluding information of non-blinded deliverers, were set to 4 or less as high RoB, 5–7 as moderate, and 8 or more as low RoB. In our study, we therefore used the cut-off: 5 or less of the 13 questions as high RoB, 6 to 8 as moderate, and 9 or more as low RoB.

### Data analysis and assessment of certainty of evidence

Meta-analyses were conducted in IBM SPSS Statistics Version 30.0.0.0 for each intervention approach if two or more studies reached sufficient methodological and statistical homogeneity [41]. Pooled and raw post-intervention data from studies were used to calculate mean differences (MDs) and standardized mean differences (SMDs) between intervention and control groups. To create forest plots with 95% confidence intervals (95% CI’s), recalculation of the data was performed if other measures of variability were used (e.g. SE) [42], or if only *P* values were available for comparisons, the Campbell Collaboration calculator (Version 2023.11.27) was used. If *P* values were not reported, the RevMan version5.3.5 calculator tool was used to recalculate means and SD, as well as CI. Random-effects model (Hedges’ g) and adjusted SE were used to acknowledge small samples and heterogeneity between studies [41], as well as non-adjusted restricted maximum likelihood (REML) for pooled pre-calculated effect size. Significance level was set to *P*<.05. All values above 50% (*I*^*2*^) heterogeneity were used for result interpretation [41]. SMDs or MDs of 0.2, 0.5, and 0.8, represent small, moderate, and large effects, respectively, while a wide 95% CI’s indicates uncertainty [43]. Each study’s contribution to the overall result was described as weight percentages [41]. To detect potential publication bias, funnel plots were produced when ≥ 5 studies were available, given that smaller numbers, interpretations are tentative [41]. If too few studies were included, databases of registered clinical trials were searched.

The Grading of Recommendations Assessment, Development, and Evaluation (GRADE) approach [44, 45] was used to evaluate the certainty of evidence (CoE) for each intervention approach and pooled separate outcomes, to present reliable findings [46]. In brief, the first step of GRADE is to decide on a starting point for the CoE, and we decided to set this at the highest possible level, as only RCTs were included in the review. The CoE was thereafter lowered with one or two grades by appraising the potential limitations (not serious; serious; very serious) due to the domains: study limitations (risk of bias), inconsistency in results (heterogeneity), indirectness (population and intervention relevance), imprecision in results (wide CIs or crossing line of no effect, heterogeneity), and publication bias (dissemination bias). The level was increased if large effects or “dose–response” relationships were detected. Finally, the findings were presented together with the certainty of the results using four levels of evidence: high (+ + + +), moderate (+ + +), low (+ +), or very low (+). Whether the CoE was supported by the studies not included in the meta-analyses, was also described. The assessment was performed independently by two reviewers (KH, WJAG) using the software GRADEpro GDT. The GRADE approach was a deviation from the PROSPERO protocol and enabled consideration of aspects outside the JBI RoB tool, such as lack of power calculation.

## Results

### Study selection

The search resulted in 4,980 records, of which 136 reports were assessed for eligibility and 27 RCTs were included. Exclusion reasons are displayed in Additional file 3.

**Fig. 1:**
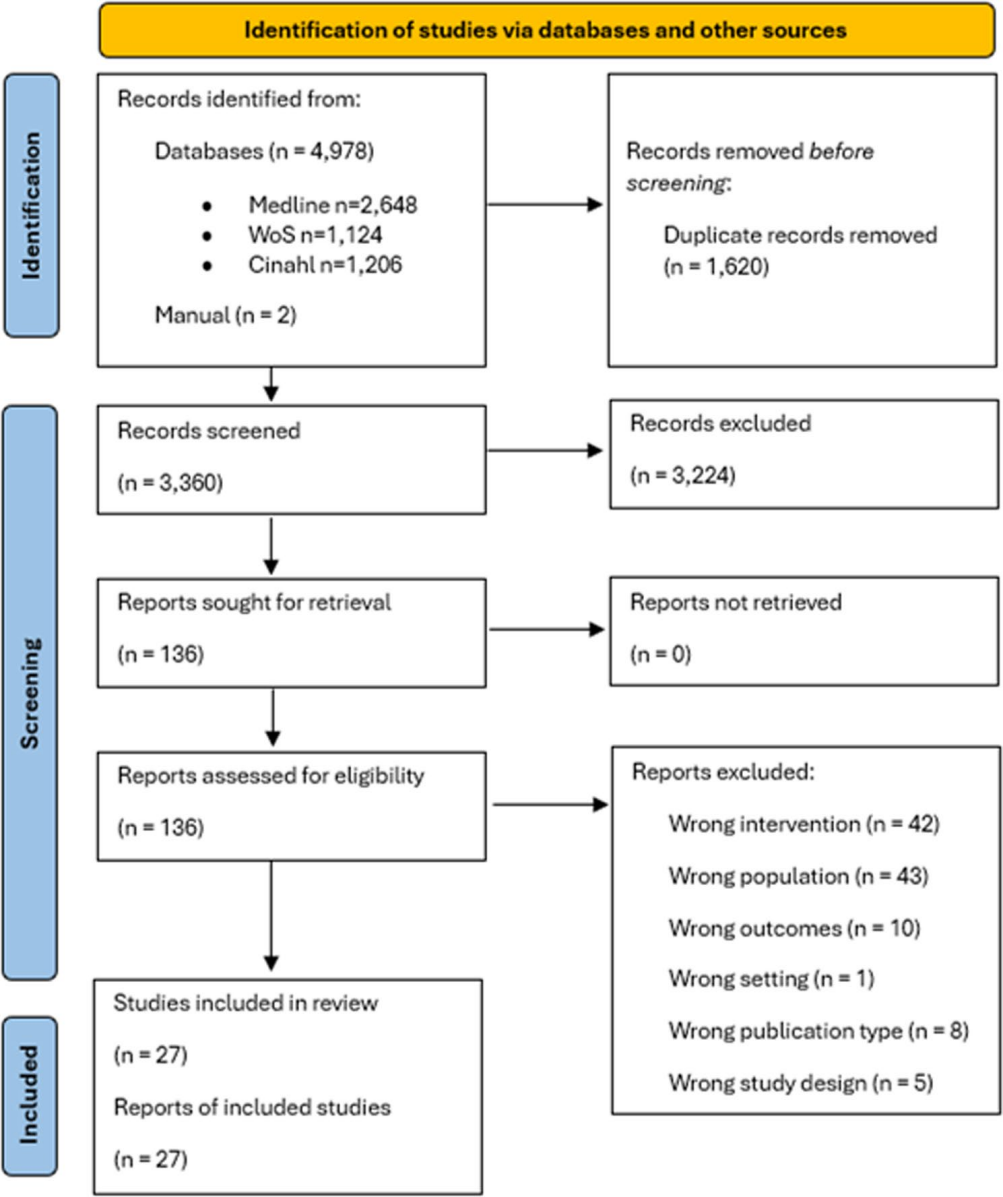
The PRISMA workflow chart [24]

The selection process is presented in Fig. 1. Twenty-two of the 27 RCTs could be pooled and were selected for the synthesis of evidence using the GRADE approach describing the evidence for intervention approaches and specific outcome measures.

### Critical appraisal

The methodological quality of the studies is presented in Table 1, grouped according to the intervention approach (see Section *Study characteristics*). Twenty-three studies achieved nine points of the JBI tool or more, describing a low RoB, three studies had moderate risk, and one study had high risk (Table 1). The most common risks of bias were the use of non-blinded participants or non-blinded therapists.

**Table 1:**
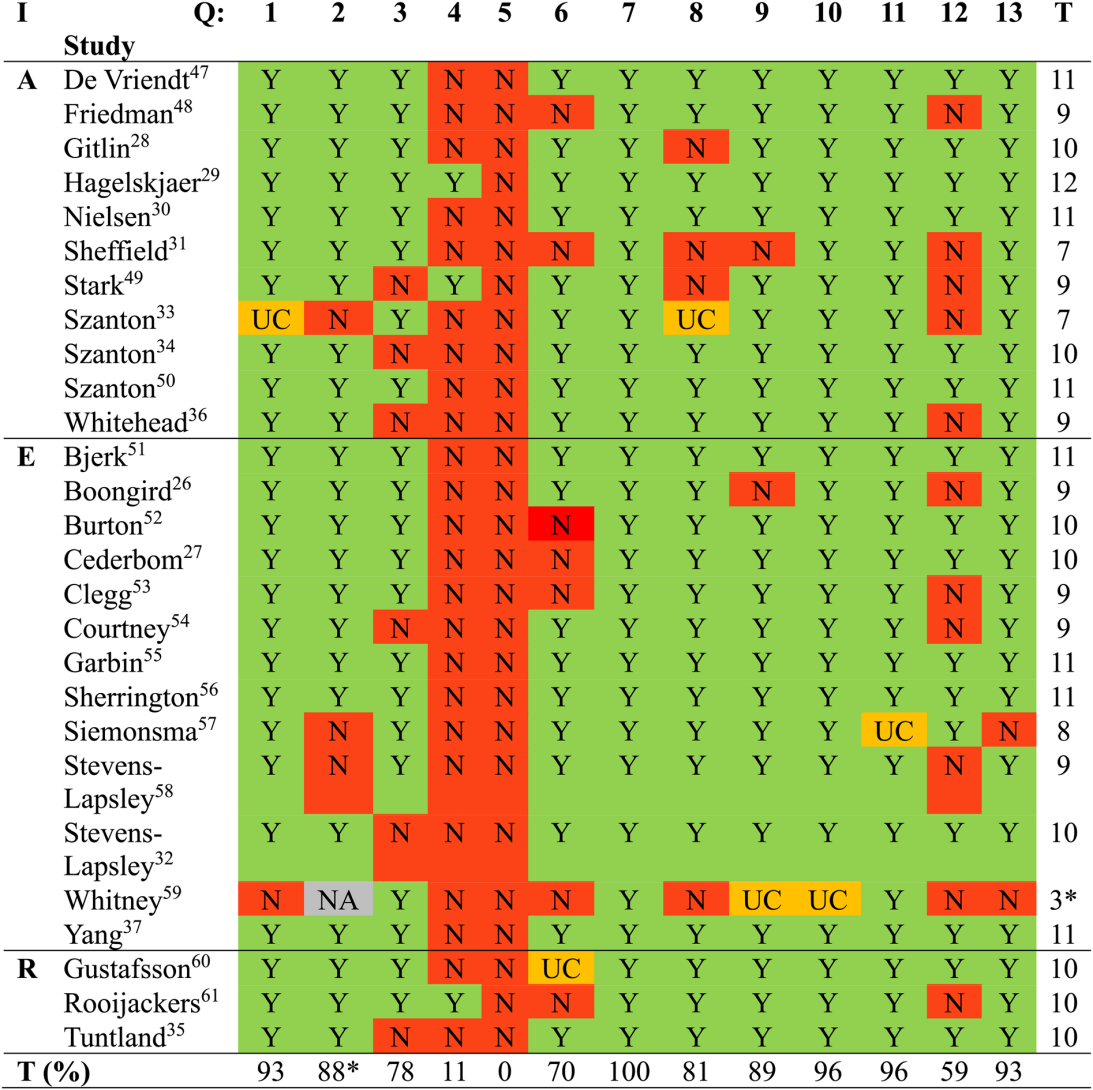
Quality assessment of included articles in the systematic review [26–36, 47–61]

JBI critical appraisal tool, question (Q) 1–13: Q1) randomization, Q2) allocation, Q3) Similarity at baseline, Q4) Blinded participants, Q5) Blinded deliverer of treatment, Q6) Blinded assessors, Q7) Groups treated identically apart from intervention, Q8) Follow up complete or described and analyzed, Q9) Participants analyzed in allocated group, Q10) Outcomes measured same way, Q11) Outcomes assessed reliably, Q12) Appropriate statistical analysis, Q13) Appropriate trial design. Possibility of bias has been addressed Y = yes, N = no, UC=unclear, NA = not applicable. Cut-off risk of bias ≤ 5 high risk, 6–8 moderate risk, > 9 low risk. *12 of 13 appraisal questions used

*Abbreviations*: *A* activity-based, *E* exercise-based, *I* intervention, *R* reablement-based, *T* total

### Study characteristics

The selected studies were grouped in reflection of the focus of their intervention approach – Activity-, Exercise-, or Reablement-based.

The Activity-based interventions included 11 studies [28–31, 33, 34, 36, 47–50], the Exercise-based consisted of 13 studies [26, 27, 32, 37, 51–59], and three studies addressed Reablement-based interventions [35, 60, 61], built on the holistic concept reablement [62, 63]. All but one study [26] was conducted in Western countries. Sources for funding were reported in all studies except one [31].

For each grouped intervention approach, the key components of the intervention were synthesized (Table 2) from what was reported in the studies, highlighting unique components for each intervention approach. Some components were represented in all intervention approaches while some were specific to the approach, all with a general focus on facilitating ADL and supporting self-training.

**Table 2:** Synthesized key components for each intervention approach

Activity-based	Exercise-based	Reablement-based
***Staff preparation components*** -Course, guided practice, follow-up meetings ***Intervention content components*** -Assessment ADL, selected ADL tasks of relevance -Personal activity goal at home/community -Motivational Interviewing -Address intrinsic, cognitive, physical, mood barriers -Strategies†: • Simplify • Prioritize • Adapt • Compensate -Practicing tasks/activities -Assistive product, environment modification -Balance and fall risk recovery technics -Strength and balance exercise -Home safety† -Medication management†	***Staff preparation components*** -Workshop exercise program ***Intervention content components*** -Physical function improvement goal -Motivational Interviewing -Environmental modification -Daily activity for training -Training physical and psychological skills to increase self-efficacy -Progressed individually tailored exercise program†: • Balance exercise • Strength exercise • Functional exercise • Stretch exercise • ADL-transfer training • Walking program • From < 1/week up to 3/day • Weights • Instruction manual • Logbook	***Staff preparation components*** -Course, kick-off meeting -Self-management ideology ***Intervention content components*** -Therapist goal assessment, training -Staff collaboration†: • Assessment to select reablement approach • Setting personal activity goal • Therapists supervise home-care service personnel • Continuous team communication - Home-care service personnel†: • Stimulate self-performed ADL • Adapt activity • Provide environment modifications • Supervise simple physical exercise program

†Unique component for the specific intervention approach. *ADL* activities of daily living

For each study, key characteristics and intervention components are presented in Additional file 4 and outcome results of ADL and physical performance in Additional files 5 to 7 (see each intervention approach section in Section *Results of syntheses*). Of the studies that reported adverse events, the following risks were described: pain or discomfort due to ankle cuffs [51], at least 48 h activity limitation due to pain [56], cut with a knife during a kitchen intervention [30], or equal amount of strains between groups [26, 32, 53, 55]. Seven studies had no adverse events to report [27, 35, 37, 48, 49, 54, 58].

### Results of syntheses

The evaluation focus differed among the included studies (Additional files 5 to 7). Studies on Activity-based interventions mainly assessed ADL using different types of ADL measurements. The studies on Exercise-based interventions most often focused on physical performance though two exclusively addressed ADL (combined BADL and IADL) [57, 59]. While, the studies on Reablement-based interventions assessed physical performance, ADL, and selected ADL tasks.

### Activity-based intervention

The participants in each study of the Activity-based interventions (*n* = 2,251), were between 75 and 83 years of age, of which 27 to 95% were women.

Key components and components for each of the 11 studies are presented in Table 2 and Additional file 4, respectively, showing the breadth of used components, except in Stark et al. [49], focusing on environmental modification only. The intervention duration and face-to-face support varied largely, from 8 weeks including between one to three home visits [47], to 24 months including monthly home visits [48], or twice visits per week for 3 months [30]. Some studies used specific intervention programs. For example, the three studies of Szanton et al. [33, 34, 50], evaluated the program Community Aging in Place Advancing Better Living for Elders (CAPABLE). Control group interventions are reported in Additional file 4.

Outcome measurements differed between studies. Some used client-centered measurements where the participant chooses ADL tasks to address AMPS (Assessment of Motor and Process Skills), COPM (Canadian Occupational Performance Measure), and I-HOPE (In-Home Occupational Performance Evaluation). The other measurements contained a wide range of BADL questions or combined BADL and IADL (FIM and ADL-I), or difficulty or dependency in performing BADL or IADL (modified WHO questionnaire, Katz-ADL Index, Barthel Index) (Additional file 5).

In seven studies [28–30, 34, 47–49], ADL improved significantly post-intervention and/or at long-term follow-up compared to control groups. BADL was improved in four studies [28, 34, 47, 48] and IADL in one study [28]. The three studies that evaluated selected ADL tasks (AMPS, COPM, and I-HOPE), showed improvement at long-term follow-up (3 months to 10 months) after completing the interventions [29, 30, 49]. One study reported moderate effect size on BADL and IADL [33]. Two studies that evaluated physical performance showed inconsistent results [28, 50] (Additional file 5).

**Fig. 2:**
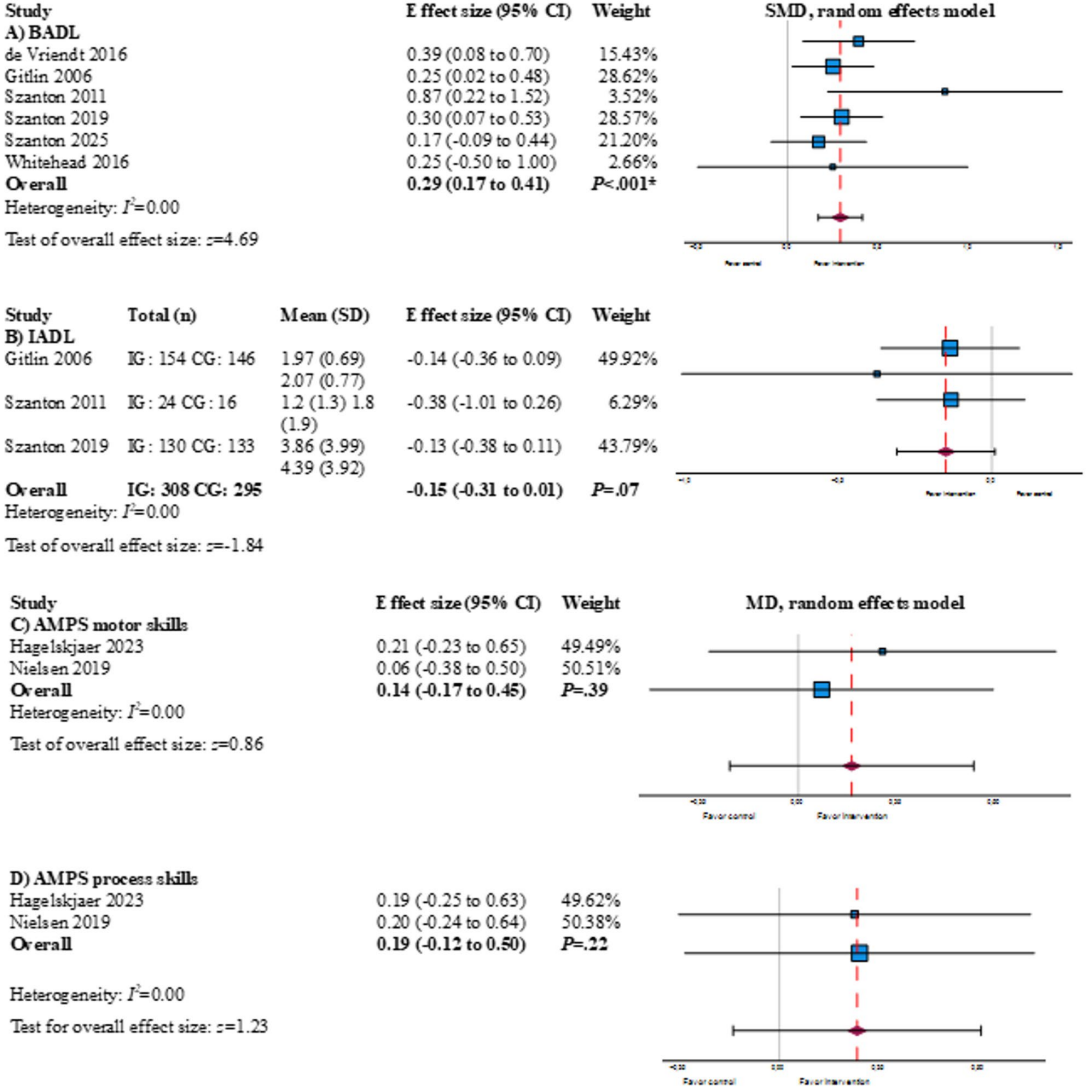
Forest Plot meta-analysis Activity-based interventions SMDs or MDs: **A**) BADL, **B**) IADL, **C**) AMPS motor skills, **D**) AMPS process skills. 95% CI. P<.05. *=significant favor IG.Abbreviations: CG=control group; IG=intervention group; n=sample size

Of the Activity-based studies, six studies (*n* = 1048) [28, 33, 34, 36, 47, 50] were included in a meta-analysis on effects on BADL. The meta-analysis showed a small statistically significant improvement for BADL in favor of the intervention (SMD = 0.29, 95% CI 0.17 to 0.41; *P*<.001) compared to control (i.e. usual care community care services or reablement or supervised sedentary activity or no intervention) (Fig. 2A). The two studies [31, 48] that were not included in the meta-analysis, showed inconsistent results. For BADL, the funnel plot showed a symmetric spread around the mean effect size (Additional file 8) indicating a low risk for publication bias. There was moderate CoE that Activity-based interventions can improve BADL; the CoE was downgraded one level due to high RoB (Table 3 in Section *Certainty of evidence*).

**Table 3:** Summary of findings

Intervention	Outcome	Risk of bias	Inconsistency	Indirectness	Impression	Publication bias	SMDs or MDs (95% CI), *P* value	Number of participants		Certainty of evidence (GRADE)
								IG	CG	
A	BADL	S^†^	NS	NS	NS	NS	0.29 (0.17 to 0.41) *P*<.001*	536	512	⊕⊕⊕◯
A	IADL	S^†^	NS	NS	S^‡^	NS	-0.15 (-0.31 to 0.01), *P*=.07	308	295	⊕⊕◯◯
A	AMPS motor skills	NS	NS	NS	S^‡^	NS	0.14 (-0.17 to 0.45), *P*=.39	74	84	⊕⊕⊕◯
A	AMPS process skills	NS	NS	NS	S^‡^	NS	0.19 (-0.12 to 0.50), *P*=.22	74	84	⊕⊕⊕◯
E	BADL	VS^#^	NS	NS	S^‡^	NS	0.43 (0.21 to 0.66), *P*<.001*	164	146	⊕◯◯◯
E	Physical performance	S^†^	NS	NS	S^‡^	NS	0.20 (0.10 to 0.30) *P*<.001*	742	730	⊕⊕◯◯
R	COPM performance	S^†^	S^§^	NS	S^‡^	NS	0.30 (-0.25 to 0.86), *P*=.28	148	143	⊕◯◯◯
R	COPM satisfaction	S^†^	NS	NS	S^‡^	NS	0.19 (-0.04 to 0.42), *P*=.10	148	143	⊕⊕◯◯
R	Physical performance	S^†^	NS	NS	S^‡^	NS	0.12 (-0.05 to 0.28), *P*=.16	281	274	⊕⊕◯◯

**Fig. 3:**
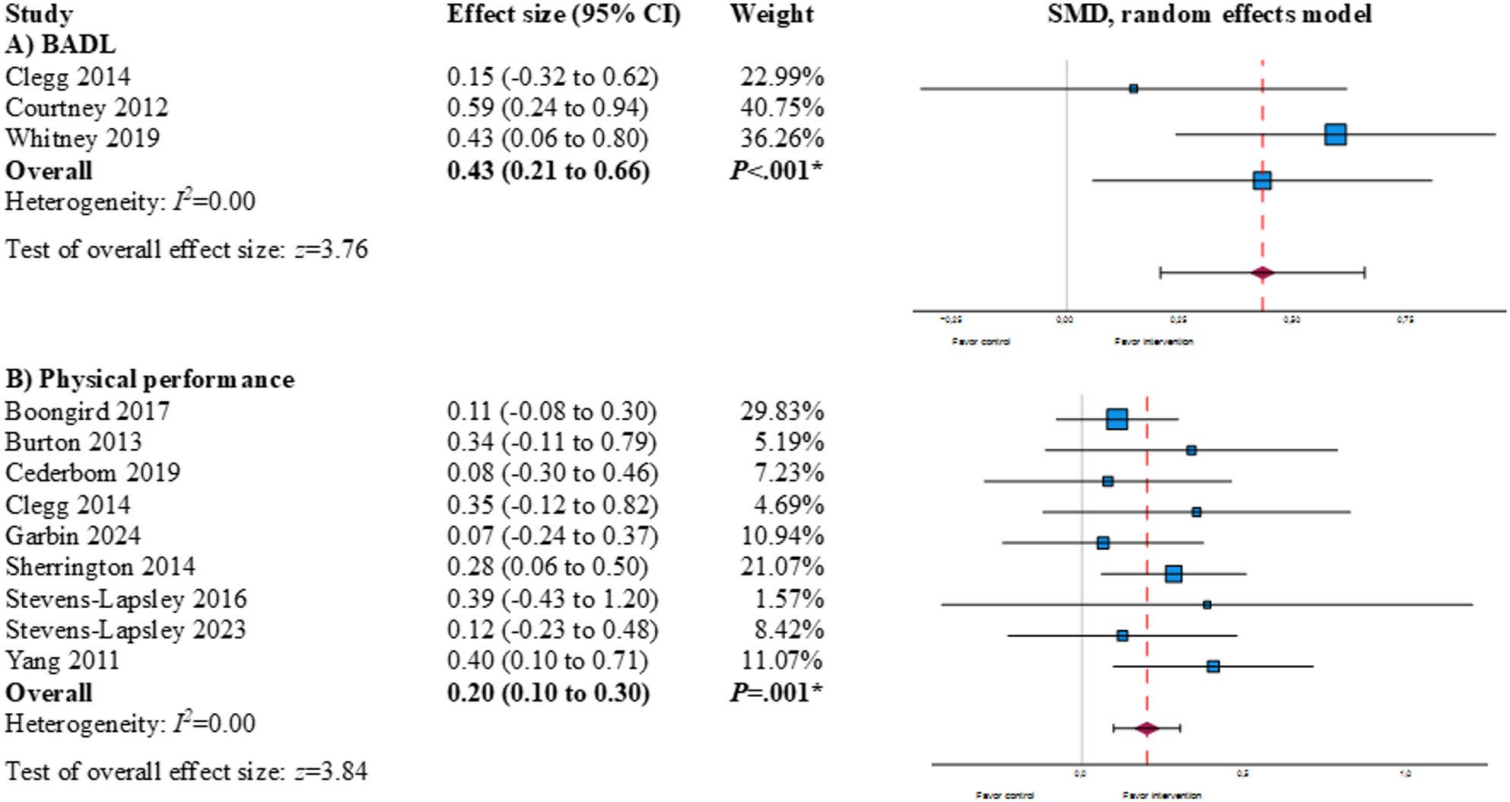
Forest Plot meta-analysis Exercise-based interventions SMDs: **A**) BADL, **B**) Physical performance. 95% CI. P<.05. *=significant favor IG

**Fig. 4:**
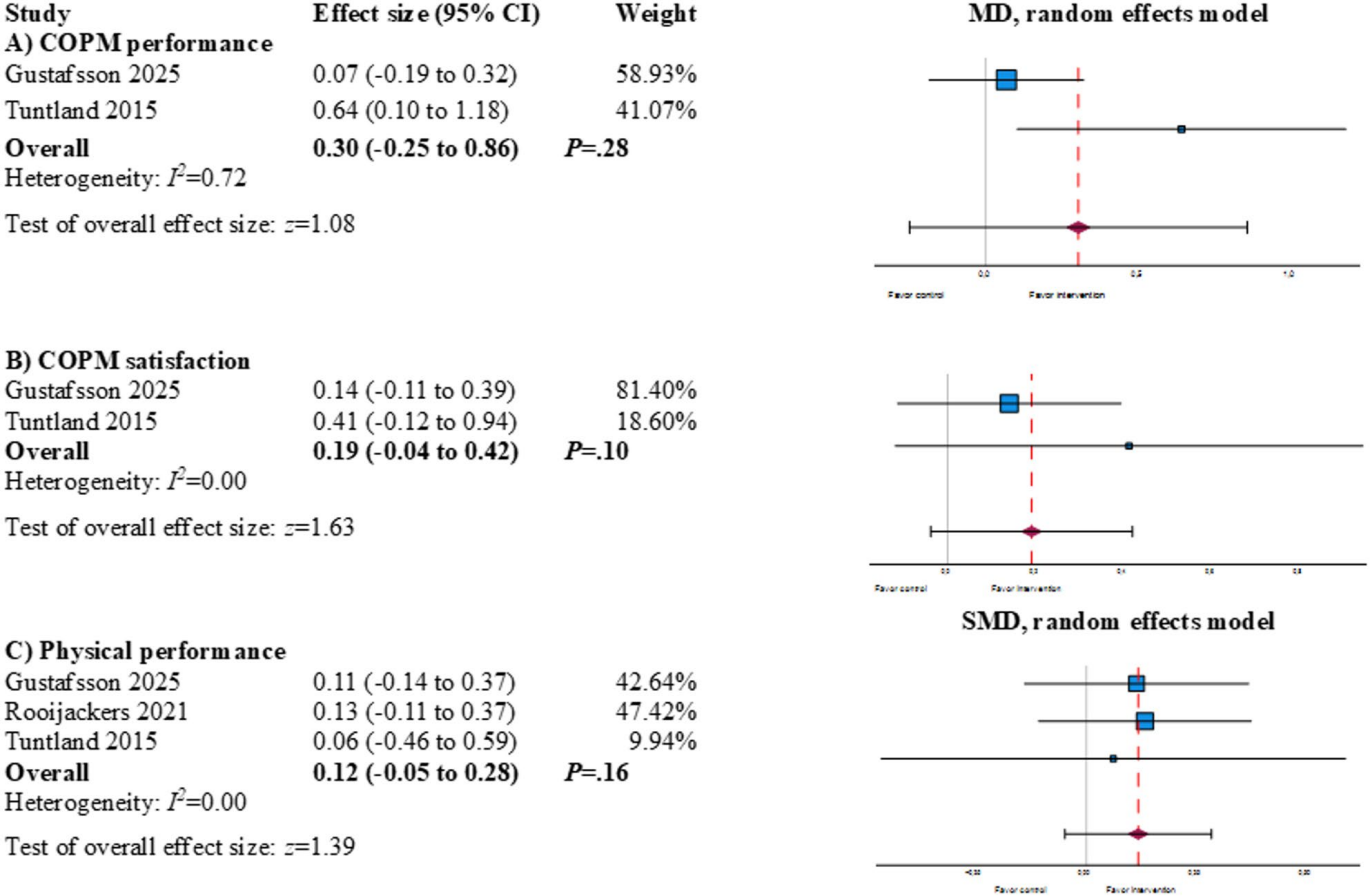
Forest Plot meta-analysis Reablement-based interventions MDs or SMDs: **A**) COPM performance, **B**) COPM satisfaction, **C**) Physical performance. 95% CI. P<.05. *=significant favor IG

Appraising potential limitations each domain: *NS* not serious, *S* serious, *VS* very serious. Downgrading domain one level due to: †=risk of bias according to the JBI tool (Table 1) and concerns regarding power calculation; ‡=CIs crossing the clinical decision threshold or wide CI in specific studies showing low precision of result (Figs. 2b-d and 3a-b, and 4a-c); §=heterogeneity above 50% (*I*^*2*^), inconsistency between the overall result and the majority of studies (Figs. 3b and 4a). Downgrading domain two levels due to: #=high risk of bias according to the JBI tool and concerns regarding power calculation (Table 1 and Additional file 6). Certainty of evidence: high (+ + + +), moderate (+ + +), low (+ +), or very low (+). 95% CI. *P*<.05. *=significant favor IG

*Abbreviations*: *A* activity-based, *CG* control group, *CI* confidence intervals, *E* exercise-based, *IG* intervention group, *MD* mean difference, *R* reablement-based, *SMD* standardized mean difference

In the meta-analysis on IADL, three of the Activity-based studies (*n* = 603) [28, 33, 34] were included. The meta-analysis did not show effect on IADL (SMD=-0.15 (95% CI -0.31 to 0.01, *P*=.07) compared to control (i.e. supervised sedentary activity or no intervention) (Fig. 2B). The non-significant outcome of Friedman et al. [48], not included in the meta-analysis, supported the result. No formal assessment of publication bias could be conducted due to the low number of included studies. However, no indications of selective reporting or missing publications were found. The CoE was low and downgraded by two levels due to high RoB and imprecision of results (Table 3 in Section *Certainty of evidence*).

Two of the Activity-based studies (*n* = 158) [29, 30] were included in the meta-analysis on selected ADL tasks (AMPS motor and process skills respectively) showing no effects (MD = 0.14 (95% CI -0.17 to 0.45, *P*=.39); MD = 0.19 (95% CI -0.12 to 0.50, *P*=.22)) compared to control (i.e. possible reablement including OT referral or municipality OT ADL tasks) (Fig. 2C-D). No formal assessment of publication bias could be conducted due to the low number of included studies. However, no indications of publication bias were found. The CoE for Activity-based interventions to affect AMPS motor skills or AMPS process skills were moderate and downgraded one level due to imprecision in results. Additional measurement tool for selected ADL tasks was used by another study [49] without reporting *P* value at first follow-up and was therefore not used to describe the CoE (Table 3 in Section *Certainty of evidence*).

### Exercise-based intervention

The participants in each study of the Exercise-based interventions (*n* = 2,135), were between 74 and 87 years of age, of which 23 to 88% were women.

Key components are presented in Table 2, and components for each of the 13 studies in Additional file 4. The components of the Exercise-based studies had a strong exercise focus, including exercise types and dosage, as well as intervention goals to increase physical capacity and functioning. Four studies utilized mutual goal setting or deciding on area of improvement of physical performance [27, 53, 54, 57]. Most studies used combinations of different progressed exercises including strengthening exercise. One study used functional exercise [27], another used transfer [57], while also combinations of walking, balance, and stretch exercise were used. In one study [52], the participants were given examples of how to embed challenging exercises in their everyday activities. The intervention duration was from one month [32, 58] up to 12 months [26], with a maximum of exercising everyday [52] or six times a week [26] or supervised training three times a week [32]. For control group intervention content, see Additional file 4.

One study that evaluated BADL and IADL showed significant positive effects compared to control group [54], while one study that evaluated BADL [53] and three studies that combined BADL and IADL measurements showed non-significant effects [56, 57, 59]. Five of 11 studies that evaluated physical performance showed significant improvement compared to control [37, 51, 54, 58] (Additional file 6), of which one study used non-objective outcome measurements [54].

Three of the Exercise-based studies (*n* = 310) [53, 54, 59] were included in the meta-analysis on BADL. The result showed statistical improvements (SMD = 0.43 (95% CI 0.21 to 0.66, *P*<.001) compared to control (i.e. usual care including discharge planning or fall prevention program) (Fig. 3A). Siemonsma et al. [57] with a moderate RoB and Sherrington et al. [56] with low RoB, not included in the meta-analysis, did not support the positive result of the meta-analysis. No formal assessment of publication bias could be conducted due to the low number of included studies. However, no indications of publication bias were found. The CoE that Exercise-based interventions can improve BADL compared to control was very low. The CoE was downgraded three levels due to very serious high RoB and imprecision of the results (Table 3 in Section *Certainty of evidence*).

Nine studies (*n* = 1,472) [26, 27, 32, 37, 52, 53, 55, 56, 58] were included in the meta-analysis of effects on physical performance using outcomes of Short Physical Performance Battery [27, 32, 55, 56, 58], Five-times Sit-To-Stand test [26, 37, 52], and Timed Up-and-Go test [53]. The meta-analysis showed statistically positive results (SMD = 0.20 (95% CI 0.10 to 0.30, *P*=.001)) compared to control (i.e., fall prevention education, usual care, official recommendations on physical activity but not on exercise, and standard unweighted supervised and unsupervised training) (Fig. 3B). Bjerk et al. [51] with low RoB, not included in the meta-analysis, supported these results. The funnel plot of the meta-analysis showed a symmetric spread around mean effect size, at least for the larger studies at the top of the plot (Additional file 9). The CoE of the effect on physical performance compared to control was also low; downgraded two levels due to high RoB and imprecision of the results (Table 3 in Section *Certainty of evidence*).

### Reablement-based intervention

The participants in each study of the Reablement-based interventions (*n* = 562), were between 80 and 83.6 years of age, of which 67.8 to 76% were women.

Key components are presented in Table 2, and components for each of the three studies in Additional file 4. The components regarding Staff preparation and team communication, varied (Additional file 4) from university courses in reablement and regular interprofessional meetings [60], a kick-off meeting followed by regular sessions and mentoring [61], to self-management courses and informal meetings [35]. The duration of the intervention varied between two to three months, with visits for two hours a week up to several visits a day. One study did not describe dosage [61]. Comparisons were made to usual care that also could include contact with PT and OT [35, 60], all evaluating both ADL and physical performance.

One study [35] showed a significant improvements effect on selected ADL tasks compared to control post-intervention (COPM performance) and at nine-months follow-up (COPM performance and satisfaction), while one reported a significant positive effect on physical performance [61] (Additional file 7).

Two of the Reablement-based studies (*n* = 291) [35, 60] were included in the meta-analysis on selected ADL tasks (Fig. 4A-B). The meta-analyses showed no significant effects on COPM performance (MD = 0.30 (95% CI, -0.25 to 0.86, *P*=.28)) compared to control, with very low CoE; the CoE was downgraded three levels due to high RoB, inconsistency, and imprecision of the results. Neither did COPM satisfaction (MD = 0.19 (95% CI, -0.04 to 0.42, *P*=.10)) show significant improvements compared to control, with low CoE, downgraded two levels due to high RoB and imprecision of the results (Table 3 in Section *Certainty of evidence*).

Three studies (*n* = 555) [35, 60, 61] were included in the meta-analysis on physical performance (Fig. 4C). Reablement-based interventions did not affect physical performance compared to control (SMD = 0.12 (95% CI -0.05 to 0.28, *P*=.16)), with low CoE, downgraded two levels due to high RoB and imprecision of the results (Table 3 in Section *Certainty of evidence*). No formal assessment of publication bias could be conducted due to the low number of included studies. However, no indications of publication bias were found.

### Certainty of evidence

In summary, according to the GRADE analysis, we conclude that there is moderate to very low evidence regarding effects of Activity-based, Exercise-based, or Reablement-based home rehabilitation on ADL (BADL, IADL, AMPS motor skills, AMPS process skills, COPM performance, COPM satisfaction) and physical performance (Table 3 in Section *Certainty of evidence*).

## Discussion

In this review, we investigated the components and the evidence for the effect of home rehabilitation on ADL and physical performance. We concluded that home rehabilitation comprises three main intervention approaches combining both unique and similar components. The meta-analyses of the Activity-based and Exercise-based intervention approaches showed small improvements in BADL and physical performance, whereas evidence for other ADL outcomes (i.e., IADL, and selected ADL tasks) and the Reablement-based intervention approach remain limited.

### Summarized intervention components

In clinical practice, all three approaches seem to be essential, as they contribute complementary focuses for enhancing older individuals’ performance in everyday life. A key component in Activity-based interventions is developing skills required to perform meaningful ADL tasks. Key components in Exercise-based interventions target improvement in physical capacity and supporting functional abilities in daily activities. In Reablement-based interventions, a key component is the emphasis on how interdisciplinary teams can stimulate self-performed ADL. Each approach incorporates both unique and similar key components. For instance, physical exercise appeared across all approaches ranging from simple exercises in the Reablement-based interventions to advanced Exercise-based interventions with individually tailored progressed multicomponent exercise programs, containing several aspects critical for transfer ability [64].

Within each rehabilitation approach, components varied in duration, frequency, and level of supervision. Evidence from prior reviews [18] suggests a dosage of two to six months of individually tailored program, including training sessions two to five times a week at an intensity of 50–80%. The large variation between the studies in our review makes it difficult to determine what dosages of the components are the most effective [65]. It seems that multiple aspects need to be taken into consideration when delivering interventions, such as intensity, frequency, duration, level of progression and supervision, as well as adherence, which makes it difficult to draw clear conclusions on the content and dosage of the program.

Home rehabilitation consists of structured processes of components [9] that were reflected in the identified key components of each intervention approach. These components could be combined and further investigated in future research. Integrating Activity-based and Exercise-based assessments may contribute to a comprehensive understanding of the individual’s intrinsic capacity, health conditions, behaviors, and environmental context [66].

At the same time, key components that strengthen self-management, support personal activity goals, and ensure continuous team communication should remain central also when the person utilizes home-care services.

### Effects on ADL and physical performance

Our study showed that home rehabilitation had varied effects on ADL and physical performance. The meta-analyses showed small positive effects on BADL and physical performance, but there were no significant effects on IADL or selected ADL tasks. The CoE for these results varied between moderate to very low, indicating that future RCTs of high quality could strengthen our results.

The literature describes a diversity of effects of home rehabilitation on ADL and physical performance. In contrast to our results, a previously conducted meta-analysis of complex multidisciplinary interventions addressed older adults at home or at clinic showed significant improvements on IADL but not on BADL [13]. In accordance with our results, Lin et al. [14] pooled the results of home-based exercise studies and showed improved ADL and physical performance after hospital discharge compared to no intervention or standard care. Moreover, physical exercise at home, in a facility, or in groups has been shown to significantly improve frail older people’s ADL, although in contrast to our results, not physical performance [67]. Also, a meta-analysis of physical exercise addressed older adults with ADL dependency showed varied effects on physical performance [68]. That study described challenges to evaluate programs that combined interventions of exercises, home safety, education, medication review, and others, as this might decrease the exercise focus [68]. Still, home rehabilitation consists of these components [9], and to develop evidence-based home rehabilitation research should evaluate the combined components. The diversity within our included studies regarding interventions, outcome tools, and statistics challenge comparability [68], as for what to be accomplished. For example, decreasing dependency in ADL could be in line with the intention to decrease home help service needs, while measuring experiences in performance difficulties could evaluate the older person’s engagement and capacity to perform the activity.

Only two of the 11 Activity-based studies evaluated physical performance, even though physical exercise or physical activity was included as a component in five [28, 33, 34, 48, 50]. Of the 13 Exercise-based studies, five evaluated ADL while the Reablement-based studies evaluated both outcomes. As home rehabilitation addresses both participation in meaningful daily activities [7] and increased capacity of physical performance to support ADL [5], we believe that both outcomes are important to motivate adherence.

### Methodological considerations

In this review, we focused on functional abilities, including studies of populations with low physical performance and ADL limitations to capture the general patient population within home rehabilitation. For this reason, we excluded articles focusing only on specific conditions (e.g., hip fracture, stroke, Alzheimer’s disease) or particular care situations (e.g., hospital transfer). Overall, few studies had this general functional-ability perspective. Previous reviews on home rehabilitation [9, 12], have concluded that research focusing on single health conditions could be problematic given that older adults commonly present with multimorbidity [9]. Yet, our synthesis of 27 studies with a functional-ability focus enabled us to categorize interventions into meaningful groups. However, some groups included as few as three studies, limiting the possibility for analysis.

Even though we attempted to perform a complete review of the scientific area, by using a thoroughly developed search strategy, studies of relevance may still not have been included.

With the intension to compare similar interventions in each meta-analysis and striving for low levels of indirectness using the GRADE approach, the included studies were grouped according to intervention purpose and components. If the studies would have been grouped differently other meta-analysis results most likely would have been generated.

While earlier reviews have incorporated studies with varying scientific quality [9, 12, 68], our intention was to achieve a high level of scientific evidence. We therefore included only peer-reviewed RCTs, as this design is best suited to determining intervention effects [25]. We also used GRADE to evaluate the CoE, which is of great importance in rehabilitation [46], enabling us to make evidence-based clinical recommendations and suggestions for future research. An additional strength of our review is that most studies had a low RoB according to the commonly used JBI tool. This tool comprises most aspects of quality, but there are no critical appraisal questions about adherence, which is an important factor for comprehending effect outcomes [68]. For example, in Cochrane’s RoB-tool, this issue is included in several items. Interestingly, of the included articles, 11 of the 13 Exercise-based studies reported adherence to the training program. Hence, we believe that our results would not be altered if another RoB tool was used.

Still, the CoE was downgraded due to poor study quality mainly concerning non-blinded participants and deliverer of treatment. Also, one estimate of the effect of the Exercise-based studies and one estimate of the Reablement-based studies are very uncertain. We suggest that further research is likely to have an important impact on the CoE.

A challenge in conducting the review was the variety of measurement tools and statistical methods. When pooling the studies it was not possible to divide into further subgroups of ADL dependency and performance difficulty, type of physical performance or subgroups of control group interventions, which might be considered as a limitation. The small number of included studies with the large methodological variations hampered the possibility of conducting sensitivity analyses. Another limitation is that most of the studies were conducted in Western countries, whereas one was conducted in Asia, decreasing the generalization to other healthcare systems. Future systematic reviews may therefore not be restricted to publications in English [69].

Further deviations from our PROSPERO protocol concern our focus on components, a change from the previous focus on mechanisms of impact, initiated from the research program Re@home, of which this systematic review is part of. Another deviation was that assessment tools outside the scope of this review were not reported. Instead, these can be found in the included studies.

### Clinical implications

To enhance BADL among older adults with low physical performance, home rehabilitation might benefit from using structured Activity-based interventions and Exercise-based interventions, which have also shown effects on physical performance. As evidence of the effects on IADL and selected ADL tasks, and the effectiveness of Reablement-based interventions are less clear, clinicians should interpret these findings cautiously and engage in ongoing evaluation of intervention outcomes.

The key components of the different intervention approaches, such as progressed training, person-centered ADL tasks, and a coordinated interdisciplinary collaboration, could be considered. However, further high-quality research on the population is required, acknowledging intervention adherence and appropriate intervention duration, intensity, and level of face-to-face supervision.

## Conclusion

The results of this systematic review and meta-analysis contribute with knowledge regarding specification of components for older people with low physical performance and/or ADL difficulties and the effects on these outcomes.

Home rehabilitation for older adults with low physical performance comprises three main approaches - Activity-based, Exercise-based, and Reablement-based interventions - each with unique and similar components.

Evidence (moderate to very low CoE) suggests small benefits for BADL and physical performance. Effects on IADL and selected ADL tasks are uncertain as are outcomes of Reablement-based interventions. High quality RCTs with clear intervention descriptions, adequate dosage, adherence monitoring, and team communication processes are needed.

## Supplementary Information

Additional file 1. PRISMA 2020 checklist.

Additional file 2. Search strategy for the three databases.

Additional file 3. List of excluded full-text reports in the screening process.

Additional file 4. Key characteristics of the included studies.

Additional file 5. Outcome results of studies of Activity-based interventions. Outcomes each study.

Additional file 6. Outcome results of studies of Exercise-based interventions. Outcomes each study.

Additional file 7. Outcome results of studies of Reablement-based interventions. Outcomes each study.

Additional file 8. Funnel plot Activity-based interventions.

Additional file 9. Funnel plot Exercise-based interventions.

## Data Availability

All data generated or analysed during this study are included in this published article [and its supplementary information files].

## Abbreviations

ADL: Activities of Daily Living
AMPS: Assessment of Motor and Process Skills
BADL: Basic ADL
CI: Confidence interval
CoE: Certainty of evidence
COPM: Canadian Occupational Performance Measure
GRADE: Grades of Recommendation Assessment, Development, and Evaluation
I-HOPE: In-Home Occupational Performance Evaluation
IADL: Instrumental ADL
JBI: the Joanna Briggs Institute
MD: Mean difference
OT: Occupational therapist
PT: Physiotherapist
RCT: Randomized controlled trial
RoB: Risk of bias
SMD: Standardized mean difference
