# Supplementary material for: The components and effects of home rehabilitation on activities of daily living and physical performance of community dwelling older people with low physical performance – a systematic review and meta-analysis of randomized controlled trials
Source: BMC Geriatr. 2026 Jun 30;26:889. doi: 10.1186/s12877-026-07887-9 (PMC13321581; doi:10.1186/s12877-026-07887-9)
Supplement: Supplementary file 2 — Additional file 2. Search strategy for the three databases. [file 12877_2026_7887_MOESM2_ESM.docx]

**Additional file 2.** Search strategy for the three databases.

1. Medline

| Interface: Ovid MEDLINE(R) and Epub Ahead of Print, In-Process & Other Non-Indexed Citations and Daily  Number of hits: 2,648  Comment: In Ovid, two or more words are automatically searched as phrases; i.e. no quotation marks are needed | Field labels   - exp/ = exploded MeSH term - / = non exploded MeSH term - .ti,ab,kf. = title, abstract and author keywords - adjx = within x words, regardless of order - * = truncation of word for alternate endings |
| --- | --- |
| Database(s): **Ovid MEDLINE(R) ALL** Search Strategy:   \| **#** \| **Searches** \| **Results** \| \| --- \| --- \| --- \| \| 1 \| Activities of daily living/ \| 78969 \| \| 2 \| Functional status/ \| 2195 \| \| 3 \| (physical function or physical functioning or functional status or functional assessment).ti,ab,kf. \| 90644 \| \| 4 \| "activities of daily living".ti,ab,kf. \| 43813 \| \| 5 \| Rehabilitation/ \| 19058 \| \| 6 \| Physical therapy modalities/ \| 43831 \| \| 7 \| Occupational therapy/ \| 16492 \| \| 8 \| exp Exercise therapy/ \| 75772 \| \| 9 \| (rehabilitat* or reable* or physical therap* or physiotherap* or occupational therap* or exercis* or physical activit* or "activities of daily living" or endurance training or resistance training).ti,ab,kf. \| 910538 \| \| 10 \| or/1-9 \| 1048653 \| \| 11 \| Home Care Services/ \| 39260 \| \| 12 \| Independent living/ \| 15781 \| \| 13 \| (home based or home care or home health care or homecare or independent living).ti,ab,kf. \| 53025 \| \| 14 \| or/11-13 \| 89530 \| \| 15 \| exp Aged/ \| 3885754 \| \| 16 \| (elder* or old age or old* adult* or old* individual* or old* patient* or old* people or old* person* or old* population*).ti,ab,kf. \| 683361 \| \| 17 \| or/15-16 \| 4126917 \| \| 18 \| randomized controlled trial.pt. \| 661626 \| \| 19 \| controlled clinical trial.pt. \| 95770 \| \| 20 \| randomi#ed.ab. \| 892415 \| \| 21 \| clinical trials as topic.sh. \| 207569 \| \| 22 \| randomly.ab. \| 487761 \| \| 23 \| trial.ti. \| 369042 \| \| 24 \| or/18-23 \| 1790608 \| \| 25 \| exp animals/ not humans.sh. \| 5457603 \| \| 26 \| 24 not 25 \| 1660605 \| \| 27 \| (review or systematic review or meta analysis).pt. \| 3904536 \| \| 28 \| (review or meta-analy* or metaanaly*).ti. \| 1048074 \| \| 29 \| or/27-28 \| 4198242 \| \| 30 \| (10 and 14 and 17 and 26) not 29 \| 3231 \| \| 31 \| limit 30 to yr="2006 - 2024" \| 2422 \| \| 32 \| 30 and (202501* or 202502* or 202503* or 202504* or 202505* or 202506* or 202507* or 202508* or 202509*).ep,ed,dp,dt. \| 271 \| \| 33 \| 31 or 32 \| 2691 \| \| 34 \| limit 33 to english language \| 2648 \| | |

2. Web of Science Core Collection

| Interface: Clarivate Analytics  Number of hits: 1,124 | Field labels   - TS/Topic = title, abstract, author keywords and Keywords Plus - NEAR/x = within x words, regardless of order - * = truncation of word for alternate endings   Note: all searches were done using the “Exact search” function in WoS |
| --- | --- |
| \| # \| Search Query \| Results \| \| --- \| --- \| --- \| \| 1 \| TS=(rehabilitat* OR reable* OR "physical therap*" OR physiotherap* OR "occupational therap*" OR exercis* OR "physical activit*" OR "endurance training" OR "resistance training") \| 1279599 \| \| 2 \| TS=("activities of daily living" or "physical function" or "physical functioning" or "functional status" or "functional assessment") \| 142724 \| \| 3 \| #2 OR #1 \| 1380930 \| \| 4 \| TS=("home based" or "home care" or "home health care" or homecare or "independent living") \| 60167 \| \| 5 \| TS=(elder* or "old age" or "old* adult*" or "old* individual*" or "old* patient*" or "old* people" or "old* person*" or "old* population*") \| 875698 \| \| 6 \| AB=(randomi?ed or randomly) OR TI=trial \| 1726685 \| \| 7 \| #6 AND #5 AND #4 AND #3 \| 1572 \| \| 8 \| #6 AND #5 AND #4 AND #3 and Review Article (Exclude – Document Types) \| 1356 \| \| 9 \| #6 AND #5 AND #4 AND #3 and Review Article (Exclude – Document Types) and English (Languages) \| 1337 \| \| 10 \| #9 Timespan: 2006-01-01 to 2025-09-08 \| 1124 \| | |

3. Cinahl

| Interface: Ebsco  Number of hits: 1,206 | Field labels   - MH+ = exploded Cinahl Heading - MH = non exploded Cinahl Heading - TI = title - AB = abstract - XB = title/abstract - Nx = within x words, regardless of order - * = truncation of word for alternate endings |
| --- | --- |
| \| S23 \| S20 NOT S21  01/01/2006 - 09/30/2025; english; \| 1206 \| \| --- \| --- \| --- \| \| S22 \| S20 NOT S21 \| 1482 \| \| S21 \| (TI review or "meta-analy*") OR (PT review or meta-analysis) \| 768152 \| \| S20 \| (S19) AND (S15) AND (S12) AND (S9) \| 1574 \| \| S19 \| (S16) OR (S17) OR (S18) \| 640222 \| \| S18 \| AB (randomi?ed or randomly) OR TI trial \| 479936 \| \| S17 \| (MH "Clinical Trials+") \| 378127 \| \| S16 \| PT randomized controlled trial \| 172413 \| \| S15 \| (S13) OR (S14) \| 1097039 \| \| S14 \| XB (elder* or "old age" or "old* adult*" or "old* individual*" or "old* patient*" or "old* people" or "old* person*" or "old* population*") \| 268273 \| \| S13 \| MH "Aged+" \| 1019912 \| \| S12 \| (S11) OR (S10) \| 52381 \| \| S11 \| XB ("home based" or "home care" or "home health care" or homecare or "independent living") \| 34950 \| \| S10 \| (MH "Home Health Care") \| 29526 \| \| S9 \| (S8) OR (S7) OR (S6) OR (S5) OR (S4) OR (S3) OR (S2) OR (S1) \| 592577 \| \| S8 \| XB (rehabilitat* OR reable* OR "physical therap*" OR physiotherap* OR "occupational therap*" OR exercis* OR "physical activit*" OR "activities of daily living" OR "endurance training" OR "resistance training") \| 412553 \| \| S7 \| XB ("activities of daily living" or "physical function" or "physical functioning" or "functional status" or "functional assessment") \| 53594 \| \| S6 \| MH "Functional Status" OR MH "Functional Assessment+" \| 64000 \| \| S5 \| (MH "Activities of Daily Living+") \| 91521 \| \| S4 \| (MH "Therapeutic Exercise+") \| 77395 \| \| S3 \| (MH "Occupational Therapy") \| 25169 \| \| S2 \| (MH "Physical Therapy") \| 42954 \| \| S1 \| (MH "Rehabilitation") \| 20787 \| | |
