# Supplementary material for: The components and effects of home rehabilitation on activities of daily living and physical performance of community dwelling older people with low physical performance – a systematic review and meta-analysis of randomized controlled trials
Source: BMC Geriatr. 2026 Jun 30;26:889. doi: 10.1186/s12877-026-07887-9 (PMC13321581; doi:10.1186/s12877-026-07887-9)
Supplement: Supplementary file 3 — Additional file 3. List of excluded full-text reports in the screening process. [file 12877_2026_7887_MOESM3_ESM.docx]

**Additional File 3.** List of excluded full-text reports in the screening process.

| **Study** | **Exclusion reason** |
| --- | --- |
| Arkkukangas M, Strömqvist Bååthe K, Ekholm A, Tonkonogi M. High Challenge Exercise and Learning Safe Landing Strategies among Community-Dwelling Older Adults: A Randomized Controlled Trial. International Journal of Environmental Research and Public Health. 2022;19(12):7370. https://dx.doi.org/10.3390/ijerph19127370 | Wrong intervention: centre-based |
| Bårdstu HB, Andersen V, Fimland MS, Aasdahl L, Lohne-Seiler H, Saeterbakken AH. Physical Activity Level Following Resistance Training in Community-Dwelling Older Adults Receiving Home Care: Results from a Cluster-Randomized Controlled Trial. International journal of environmental research and public health. 2021;18(13):6682. doi: 10.3390/ijerph18136682 | Wrong intervention: centre-based |
| Bårdstu HB, Andersen V, Fimland MS, Aasdahl L, Raastad T, Cumming KT, et al. Effectiveness of a resistance training program on physical function, muscle strength, and body composition in community-dwelling older adults receiving home care: a cluster-randomized controlled trial. European Review of Aging and Physical Activity. 2020;17(11).  https://dx.doi.org/10.1186/s11556-020-00243-9 | Wrong intervention: centre-based |
| Boa Sorte Silva NC, Gill DP, Gregory MA, Bocti J, Petrella RJ. Multiple-modality exercise and mind-motor training to improve mobility in older adults: A randomized controlled trial. Experimental gerontology. 2018;103:17–26. doi: 10.1016/j.exger.2017.12.011 | Wrong intervention: centre-based |
| Brach JS, Perera S, Shuman V, Gil AB, Kriska A, Nadkarni NK, et al. Effect of Timing and Coordination Training on Mobility and Physical Activity Among Community-Dwelling Older Adults: A Randomized Clinical Trial. JAMA network open. 2022;5(5):e2212921. doi: 10.1001/jamanetworkopen.2022.12921 | Wrong intervention: centre-based |
| Chi Y-C, Wu C-L, Liu H-T. Effect of a multi-disciplinary active aging intervention among community elders. Medicine (Baltimore). 2021;100(51):e28314–e28314. doi: 10.1097/MD.0000000000028314 | Wrong intervention: centre-based |
| Costilla M, Corral-Pérez J, Vázquez-Sánchez MÁ, Ávila-Cabeza-de-Vaca L, González-Mariscal A, Casals C. Improvements in Depressive Symptoms, Perceived Social Support, and Quality of Life Through an Educational Program in Community-Dwelling Older Adults With Frailty Phenotype: A Randomized Controlled Trial of the FRAGSALUD Project. The American journal of geriatric psychiatry. 2025;33(8):877–90. doi: 10.1016/j.jagp.2025.02.014 | Wrong intervention: centre-based |
| Danilovich M, Corcos D, Eisenstein, A, Marquez D, Hughes S. The Impact of Strong for Life on the Physical Functioning and Health of Older Adults Receiving Home and Community-Based Services. Aging and Society. 2017;7(2):1-10. https://dx.doi.org/10.18848/2160-1909/CGP/v07i02/1-10 | Wrong intervention: centre-based |
| Duan Y, Yang M, Wang Y, Cheng S, Liang W, Lippke S, et al. Effects of a blended face-to-face and eHealth lifestyle intervention on physical activity, diet, and health outcomes in Hong Kong community-dwelling older adults: a randomized controlled trial. BMC public health. 2025;25(1). doi: 10.1186/s12889-025-23311-0 | Wrong intervention: centre-based |
| Gallo E, Stelmach M, Frigeri F, Ahn D-H. Determining Whether a Dosage-Specific and Individualized Home Exercise Program With Consults Reduces Fall Risk and Falls in Community-Dwelling Older Adults With Difficulty Walking: A Randomized Control Trial. Journal of geriatric physical therapy (2001). 2018;41(3):161–72. doi: 10.1519/JPT.0000000000000114 | Wrong intervention: centre-based |
| Jiang G, Tan X, Zou J, Wu X. A 24-Week Combined Resistance and Balance Training Program Improves Physical Function in Older Adults: A Randomized Controlled Trial. Journal of Strength and Conditioning Research. 2025;39(1):e62–9. https://dx.doi.org/10.1519/JSC.0000000000004941 | Wrong intervention: centre-based |
| Kim H, Won CW, Kim M, Kojima N, Fujino K, Osuka Y, et al. The effects of exercise and milk-fat globule membrane (MFGM) on walking parameters in community-dwelling elderly Japanese women with declines in walking ability: A randomized placebo controlled trial. Archives of Gerontology and Geriatrics. 2019;83:106–13. https://dx.doi.org/10.1016/j.archger.2019.03.029 | Wrong intervention: centre-based |
| Li Y, Li S, Weng X, Yang X, Bao J, Liao S, et al. Effects of the Vivifrail-B multicomponent exercise program based on society ecosystems theory on physical function in community-dwelling frail older adults: A randomized controlled trial. Experimental Gerontology. 2025;200. https://dx.doi.org/10.1016/j.exger.2024.112670 | Wrong intervention: centre-based |
| Liu C ju, Xu H, Keith N, Clark D. Promoting ADL independence in vulnerable, community-dwelling older adults: a pilot RCT comparing 3-Step Workout for Life versus resistance exercise. Clinical Interventions in Aging. 2017;12:1141–9. https://dx.doi.org/10.2147/CIA.S136678 | Wrong intervention: centre-based |
| Rössler R, Birrer M, Haslbauer A, Goldfarb J, De Spiegeleer A, Härdi I, et al. Efficacy of the power centering for seniors intervention on physical functional performance in older community-dwelling adults: a secondary analysis of a randomised controlled trial. Scientific Reports. 2025;15(1). https://doi.org/10.1038/s41598-025-13404-6 | Wrong intervention: centre-based |
| Saeterbakken AH, Bårdstu HB, Brudeseth A, Andersen V. Effects of Strength Training on Muscle Properties, Physical Function, and Physical Activity among Frail Older People: A Pilot Study. Grosset JF, editor. Journal of Aging Research. 2018;2018(2018):1–11. https://dx.doi.org/10.1155/2018/8916274 | Wrong intervention: centre-based |
| Shinji Hattori, Toshiyuki Yoshida, Yasuyuki Okumura, Katsunori Kondo. Effects of Reablement on the Independence of Community-Dwelling Older Adults with Mild Disability: A Randomized Controlled Trial. International Journal of Environmental Research and Public Health. 2019;16:3954. doi: 10.3390/ijerph16203954 | Wrong intervention: centre-based |
| Spoorenberg SLW, Wynia K, Uittenbroek RJ, Kremer HPH, Reijneveld SA. Effects of a population-based, person-centred and integrated care service on health, wellbeing and self-management of community-living older adults: A randomised controlled trial on Embrace. PloS one. 2018;13(1):e0190751. https://dx.doi.org/10.1371/journal.pone.0190751 | Wrong intervention: centre-based |
| van Dongen EJI, Doets EL, de Groot LCPGM, Dorhout BG, Haveman-Nies A. Process Evaluation of a Combined Lifestyle Intervention for Community-Dwelling Older Adults: ProMuscle in Practice. The Gerontologist. 2020;60(8):1538–54. doi: 10.1093/geront/gnaa027 | Wrong intervention: centre-based |
| Watson SL, Weeks BK, Weis LJ, Horan SA, Beck BR. Heavy resistance training is safe and improves bone, function, and stature in postmenopausal women with low to very low bone mass: novel early findings from the LIFTMOR trial. Osteoporosis international. 2015;26(12):2889–94. doi: 10.1007/s00198-015-3263-2 | Wrong intervention: centre-based |
| Wollesen B, Mattes K, Schulz S, Bischoff LL, Seydell L, Bell JW, et al. Effects of Dual-Task Management and Resistance Training on Gait Performance in Older Individuals: A Randomized Controlled Trial. Frontiers in aging neuroscience. 2017;9:415. doi: 10.3389/fnagi.2017.00415 | Wrong intervention: centre-based |
| Baba Y, Ooyama C, Tazawa Y, Kohzuki M. Effects of Adachi Rehabilitation Programme on older adults under long-term care: A multi-centre controlled trial. PloS one. 2021;16(2):e0245646. https://dx.doi.org/10.1371/journal.pone.0245646 | Wrong intervention: community-based |
| Yoo H, Chung E, Lee B-H. The Effects of Augmented Reality-based Otago Exercise on Balance, Gait, and Falls Efficacy of Elderly Women. Journal of Physical Therapy Science. 2013;25(7):797–801. doi: 10.1589/jpts.25.797 | Wrong intervention: augmented reality-based |
| Saito Y, Nakamura S, Kasukawa T, Nagasawa M, Oguma Y, Narimatsu H. Efficacy of exercise with the hybrid assistive limb lumbar type on physical function in mobility-limited older adults: A 5-week randomized controlled trial. Experimental Gerontology. 2024;195. https://dx.doi.org/10.1016/j.exger.2024.112536 | Wrong intervention: cyborg (exoskeleton) |
| Alizadeh-Khoei M, Fadayevatan R, Sharif F, Chehrehgosha M, Aminalroaya R. Effects of hospital-to-home transitional care on health outcomes of elderly patients in Islamic Republic of Iran. Eastern Mediterranean Health Journal. 2023;29(6):451–61. https://dx.doi.org/10.26719/emhj.23.042 | Wrong intervention: no OT or PT intervention |
| Areán PA, Raue PJ, McCulloch C, Kanellopoulos D, Seirup JK, Banerjee S, et al. Effects of Problem-Solving Therapy and Clinical Case Management on Disability in Low-Income Older Adults. The American Journal of Geriatric Psychiatry. 2015;23(12):1307–14. https://dx.doi.org/10.1016/j.jagp.2015.04.005 | Wrong intervention: no OT or PT intervention |
| Buurman BM, Parlevliet JL, Allore HG, Blok W, van Deelen BAJ, Moll van Charante EP, et al. Comprehensive Geriatric Assessment and Transitional Care in Acutely Hospitalized Patients: The Transitional Care Bridge Randomized Clinical Trial. JAMA Internal Medicine. 2016;176(3):302–9. https://dx.doi.org/10.1001/jamainternmed.2015.8042 | Wrong intervention: no OT or PT intervention |
| Cerrah L, Oral M, Umar EK, Pak M. Enhancing the quality of life of older people in rural Türkiye: A randomised controlled appraisal of solution‐focused case management. Australasian Journal on Ageing. 2025;44(1):1-10. 10.1111/ajag.13406 | Wrong intervention: no OT or PT intervention |
| Hoogendijk EO, van der Horst HE, van de Ven PM, Twisk JWR, Deeg DJH, Frijters DHM, et al. Effectiveness of a Geriatric Care Model for frail older adults in primary care: Results from a stepped wedge cluster randomized trial. European Journal of Internal Medicine. 2016;28:43–51. https://dx.doi.org/10.1016/j.ejim.2015.10.023 | Wrong intervention: no OT or PT intervention |
| Howel D, Moffatt S, Haighton C, Bryant A, Becker F, Steer M, et al. Does domiciliary welfare rights advice improve health-related quality of life in independent-living, socio-economically disadvantaged people aged ≥60 years? Randomised controlled trial, economic and process evaluations in the North East of England. PloS one. 2019;14(1):e0209560. https://dx.doi.org/10.1371/journal.pone.0209560 | Wrong intervention: no OT or PT intervention |
| Lewin G, Allan J, Patterson C, Knuiman M, Boldy D, Hendrie D. A comparison of the home-care and healthcare service use and costs of older Australians randomised to receive a restorative or a conventional home-care service. Health & Social Care in the community. 2014;22(3):328–36. https://dx.doi.org/10.1111/hsc.12092 | Wrong intervention: no OT or PT intervention |
| Parsons JGM, Sheridan N, Rouse P, Robinson E, Connolly M. A Randomized Controlled Trial to Determine the Effect of a Model of Restorative Home Care on Physical Function and Social Support Among Older People. Archives of Physical Medicine and Rehabilitation. 2013;94(6):1015–22. https://dx.doi.org/10.1016/j.apmr.2013.02.003 | Wrong intervention: no OT or PT intervention |
| Ploeg J, Brazil K, Hutchison B, Kaczorowski J, Dalby DM, Goldsmith CH, et al. Effect of preventive primary care outreach on health related quality of life among older adults at risk of functional decline: randomised controlled trial. BMJ. 2010;340(7752):904–904. https://dx.doi.org/10.1136/bmj.c1480 | Wrong intervention: no OT or PT intervention |
| Vestergaard S, Kronborg C, Puggaard L. Home-based video exercise intervention for community-dwelling frail older women: a randomized controlled trial. Aging Clinical and Experimental Research. 2008;20(5):479–86. | Wrong intervention: no OT or PT intervention |
| Aoki K, Sakuma M, Endo N. The impact of exercise and vitamin D supplementation on physical function in community-dwelling elderly individuals: A randomized trial. Journal of Orthopaedic Science. 2018;23(4):682-687. https://dx.doi.org/10.1016/j.jos.2018.03.011 | Wrong intervention: no supervision at home |
| Brovold T, Skelton DA, Bergland A. Older Adults Recently Discharged from the Hospital: Effect of Aerobic Interval Exercise on Health-Related Quality of Life, Physical Fitness, and Physical Activity. Journal of the American Geriatrics Society (JAGS). 2013;61(9):1580–5. https://dx.doi.org/10.1111/jgs.12400 | Wrong intervention: no supervision at home |
| Carvalho LP, Kergoat MJ, Bolduc A, Aubertin-Leheudre M. A Systematic Approach for Prescribing Posthospitalization Home-Based Physical Activity for Mobility in Older Adults: The PATH Study. Journal of the American Medical Directors Association. 2019;20(10):1287–93. https://dx.doi.org/10.1016/j.jamda.2019.01.143 | Wrong intervention: no supervision at home |
| Hinrichs T, Bücker B, Klaaßen-Mielke R, Brach M, Wilm S, Platen P, et al. Home-Based Exercise Supported by General Practitioner Practices: Ineffective in a Sample of Chronically Ill, Mobility-Limited Older Adults (the HOMEfit Randomized Controlled Trial). Journal of the American Geriatrics Society (JAGS). 2016;64(11):2270–9. https://dx.doi.org/10.1111/jgs.14392 | Wrong intervention: no supervision at home |
| Liang IJ, Perkin OJ, Williams S, McGuigan PM, Thompson D, Western MJ. The Efficacy of 12-Week Progressive Home-Based Strength and Tai-Chi Exercise Snacking in Older Adults: A Mixed-Method Exploratory Randomised Control Trial. The Journal of Frailty & Aging. 2024;13(4):572–81. https://dx.doi.org/10.14283/jfa.2024.32 | Wrong intervention: no supervision at home |
| McAuley E, Wojcicki TR, Gothe NP, Mailey EL, Szabo AN, Fanning J, et al. Effects of a DVD-Delivered Exercise Intervention on Physical Function in Older Adults. The Journals of Gerontology Series A, Biological Sciences and Medical Sciences. 2013;68(9):1076–82. https://dx.doi.org/10.1093/gerona/glt014 | Wrong intervention: no supervision at home |
| Wójcicki TR, Fanning J, Awick EA, Olson EA, Motl RW, McAuley E. Maintenance Effects of a DVD-Delivered Exercise Intervention on Physical Function in Older Adults. The Journals of Gerontology Series A, Biological Sciences and Medical Sciences. 2015;70(6):785–9. https://dx.doi.org/10.1093/gerona/glu188 | Wrong intervention: no supervision at home |
| Zhou J, Liu B, Xu J, Wang F, Ye H, Duan J-P, et al. Home-based strength and balance exercises for fall prevention among older individuals of advanced age: a randomized controlled single-blind study. Annals of medicine (Helsinki). 2025;57(1):2459818. doi: 10.1080/07853890.2025.2459818 | Wrong intervention: no supervision at home |
| Bankole AO, Zhang Y, Hu D, Preisser JS, Colón-Emeric C, Toles M. Life-Space of Older Adults after Discharge from Skilled Nursing Facilities. Journal of the American Medical Directors Association. 2024;25(7). 10.1016/j.jamda.2024.01.006 | Wrong outcome: |
| Breysse J, Dixon S, Wilson J, Szanton S. Aging Gracefully in Place: An Evaluation of the Capability of the CAPABLE© Approach. Journal of Applied Gerontology. 2022;41(3):718–28. 10.1177/07334648211042606 | Wrong outcome: do not reach criteria for inclusion |
| Hagelskjær V, Nielsen KT, von Bülow C, Graff M, Wæhrens EE. Occupational therapy addressing the ability to perform activities of daily living among persons living with chronic conditions: a randomised controlled pilot study of ABLE 2.0. Pilot and Feasibility Studies. 2021;7(1). 10.1186/s40814-021-00861-9 | Wrong outcome: do not reach criteria for inclusion |
| Liu M, Xue Q, Gitlin LN, Wolff JL, Guralnik J, Leff B, et al. Disability Prevention Program Improves Life‐Space and Falls Efficacy: A Randomized Controlled Trial. Journal of the American Geriatrics Society (JAGS). 2021;69(1):85–90. https://dx.doi.org/10.1111/jgs.16808 | Wrong outcome: do not reach criteria for inclusion |
| Markle-Reid M, McAiney C, Fisher K, Ganann R, Gauthier AP, Heald-Taylor G, et al. Effectiveness of a nurse-led hospital-to-home transitional care intervention for older adults with multimorbidity and depressive symptoms: A pragmatic randomized controlled trial. PloS one. 2021;16(7):e0254573. https://dx.doi.org/10.1371/journal.pone.0254573 | Wrong outcome: do not reach criteria for inclusion |
| Parker S, Oliver P, Pennington M, Bond J, Jagger C, Enderby P, et al. Rehabilitation of older patients: day hospital compared with rehabilitation at home. A randomised controlled trial. Health Technology Assessment (Winchester, England). 2009;13(39):1–143. https://dx.doi.org/10.3310/hta13390 | Wrong outcome: do not reach criteria for inclusion |
| Parker SG, Oliver P, Pennington M, Bond J, Jagger C, Enderby P, et al. Rehabilitation of older patients: day hospital compared with rehabilitation at home. Clinical outcomes. Age and Ageing. 2011;40(5):557–62. https://dx.doi.org/10.1093/ageing/afr046 | Wrong outcome: do not reach criteria for inclusion |
| Sanford JA, Griffiths PC, Richardson P, Hargraves K, Butterfield T, Hoenig H. The Effects of In-Home Rehabilitation on Task Self-Efficacy in Mobility-Impaired Adults: A Randomized Clinical Trial. Journal of the American Geriatrics Society (JAGS). 2006;54(11):1641–8. https://dx.doi.org/10.1111/j.1532-5415.2006.00913.x | Wrong outcome: do not reach criteria for inclusion |
| Li Y, Liebel DV, Friedman B. An investigation into which individual instrumental activities of daily living are affected by a home visiting nurse intervention. Age and Ageing. 2013;42(1):27–33. https://dx.doi.org/10.1093/ageing/afs151 | Wrong outcome: single item of IADLs |
| Friedman B, Li Y, Liebel DV, Powers BA. Effects of a home visiting nurse intervention versus care as usual on individual activities of daily living: a secondary analysis of a randomized controlled trial. BMC Geriatrics. 2014;14(1). https://dx.doi.org/10.1186/1471-2318-14-24 | Wrong outcome: single items of IADLs |
| Arena SK, Wilson CM, Boright L, Peterson E. Impact of the HOP-UP-PT program on older adults at risk to fall: a randomized controlled trial. BMC geriatrics. 2021;21(1). doi: 10.1186/s12877-021-02450-0 | Wrong population: do not reach criteria for inclusion |
| Arkkukangas M, Söderlund A, Eriksson S, Johansson AC. Fall Preventive Exercise With or Without Behavior Change Support for Community-Dwelling Older Adults: A Randomized Controlled Trial With Short-Term Follow-up. Journal of Geriatric Physical Therapy (2001). 2019;42(1):9–17. 10.1519/JPT.0000000000000129 | Wrong population: do not reach criteria for inclusion |
| Bailey DP, Harper JH, Kilbride C, McGowan LJ, Victor C, Brierley ML, et al. The frail-LESS (LEss sitting and sarcopenia in frail older adults) remote intervention to improve sarcopenia and maintain independent living via reductions in sedentary behaviour: findings from a randomised controlled feasibility trial. BMC Geriatrics. 2024;24(1). https://dx.doi.org/10.1186/s12877-024-05310-9 | Wrong population: do not reach criteria for inclusion |
| Bernocchi P, Giordano A, Pintavalle G, Galli T, Ballini Spoglia E, Baratti D, et al. Feasibility and Clinical Efficacy of a Multidisciplinary Home-Telehealth Program to Prevent Falls in Older Adults: A Randomized Controlled Trial. Journal of the American Medical Directors Association. 2019;20(3):340–6. doi: 10.1016/j.jamda.2018.09.003 | Wrong population: do not reach criteria for inclusion |
| Burke L, Lee AH, Jancey J, Xiang L, Kerr DA, Howat PA, et al. Physical activity and nutrition behavioural outcomes of a home-based intervention program for seniors: a randomized controlled trial. The international journal of behavioral nutrition and physical activity. 2013;10(1):14–14. doi: 10.1186/1479-5868-10-14 | Wrong population: do not reach criteria for inclusion |
| Chang Y-H, Hung C-C, Chiang Y-Y, Chen C-Y, Liao L-C, Ma MH-M, et al. Effects of osteoporosis treatment and multicomponent integrated care on intrinsic capacity and happiness among rural community-dwelling older adults: the Healthy Longevity and Ageing in Place (HOPE) randomised controlled trial. Age and ageing. 2025;54(2). doi: 10.1093/ageing/afaf017 | Wrong population: do not reach criteria for inclusion |
| Chen SW, Krauss MJ, Somerville E, Holden B, Devine M, Stark S. Removing environmental barriers to independent living: A feasibility randomised controlled trial targeting people ageing with long-term physical disabilities. The British Journal of Occupational Therapy. 2025;88(1):5–16. 10.1177/03080226241280803 | Wrong population: do not reach criteria for inclusion |
| Cheng H, Shi M, Pu F. Construction of Fall Prevention Exercise Training Scheme for Elderly Discharged Patients Using Self-Efficacy Theory Framework. Alternative Therapies in Health and Medicine. 2024;30(2):56-63. | Wrong population: do not reach criteria for inclusion |
| Clemson L, Fiatarone Singh MA, Bundy A, Cumming RG, Manollaras K, O’Loughlin P, et al. Integration of balance and strength training into daily life activity to reduce rate of falls in older people (the LiFE study): randomised parallel trial. BMJ (Online). 2012;345(7870):14–14. https://dx.doi.org/10.1136/bmj.e4547 | Wrong population: do not reach criteria for inclusion |
| Clemson L, Singh MF, Bundy A, Cumming RG, Weissel E, Munro J, et al. LiFE Pilot Study: A randomised trial of balance and strength training embedded in daily life activity to reduce falls in older adults. Australian occupational therapy journal. 2010;57(1):42–50. doi: 10.1111/j.1440-1630.2009.00848.x | Wrong population: do not reach criteria for inclusion |
| Counsell SR, Callahan CM, Clark DO, Tu W, Buttar AB, Stump TE, et al. Geriatric Care Management for Low-Income Seniors: A Randomized Controlled Trial. JAMA : the Journal of the American Medical Association. 2007;298(22):2623–33. https://dx.doi.org/10.1001/jama.298.22.2623 | Wrong population: do not reach criteria for inclusion |
| de Carvalho Bastone A, Nobre LN, de Souza Moreira B, Rosa IF, Ferreira GB, Santos DDL, et al. Independent and combined effect of home-based progressive resistance training and nutritional supplementation on muscle strength, muscle mass and physical function in dynapenic older adults with low protein intake: A randomized controlled trial. Archives of Gerontology and Geriatrics. 2020;89. https://dx.doi.org/10.1016/j.archger.2020.104098 | Wrong population: do not reach criteria for inclusion |
| De La Vega-Cordero EM, Lopez-Teros M, Garcia-Gonzalez AI, Rosas-Carrasco O, Castillo-Aragon A. Effectiveness of an online multicomponent physical exercise intervention on the physical performance of community-dwelling older adults: A randomized controlled trial. Geriatric Nursing (New York). 2023;54:83–93. https://dx.doi.org/10.1016/j.gerinurse.2023.08.018 | Wrong population: do not reach criteria for inclusion |
| Dorresteijn TAC, Zijlstra GAR, Ambergen AW, Delbaere K, Vlaeyen JWS, Kempen GIJM. Effectiveness of a home-based cognitive behavioral program to manage concerns about falls in community-dwelling, frail older people: results of a randomized controlled trial. BMC Geriatrics. 2016;16(1). https://dx.doi.org/10.1186/s12877-015-0177-y | Wrong population: do not reach criteria for inclusion |
| Eckert T, Wronski P, Bongartz M, Ullrich P, Abel B, Kiss R, et al. Cost-Effectiveness and Cost-Utility of a Home-Based Exercise Program in Geriatric Patients with Cognitive Impairment. Gerontology (Basel). 2021;67(2):220–32. doi: 10.1159/000512748 | Wrong population: do not reach criteria for inclusion |
| Farag I, Howard K, Hayes AJ, Ferreira ML, Lord SR, Close JT, et al. Cost-effectiveness of a Home-Exercise Program Among Older People After Hospitalization. Journal of the American Medical Directors Association. 2015;16(6):490–6. https://dx.doi.org/10.1016/j.jamda.2015.01.075 | Wrong population: do not reach criteria for inclusion |
| Fisher KL, Reeder BA, Harrison EL, Bruner BG, Ashworth NL, Pahwa P et al. Comparing Class-Based and Home-Based Exercise for Older Adults With Chronic Health Conditions: 12-Month Follow-Up of a Randomized Clinical Trial. Journal of Aging and Physical Activity 2018;26(3):471-485. https://dx.doi.org/10.1123/japa.2016-0285 | Wrong population: do not reach criteria for inclusion |
| Fritz H, Hu YL. Habit Formation Intervention to Reduce Frailty Risk Factors: A Feasibility Study. The American Journal of Occupational Therapy. 2022;76(3). https://dx.doi.org/10.5014/ajot.2022.045948 | Wrong population: do not reach criteria for inclusion |
| Fugazzaro S, Schiavi M, Bucciarelli V, Formisano D, Pellegrini M, Costi S. Occupational Therapy in Complex Patients: A Pilot Randomized Controlled Trial. Mackenzie L, editor. Occupational Therapy International. 2018;2018(2018):1–11. https://dx.doi.org/10.1155/2018/3081094 | Wrong population: do not reach criteria for inclusion |
| Geraedts HAE, Dijkstra H, Zhang W, Ibarra F, Far IK, Zijlstra W, et al. Effectiveness of an individually tailored home-based exercise rogramme for pre-frail older adults, driven by a tablet application and mobility monitoring: a pilot study. European review of aging and physical activity. 2021;18(1):10. doi: 10.1186/s11556-021-00264-y | Wrong population: do not reach criteria for inclusion |
| Grant D, Tomlinson D, Tsintzas K, Kolić P, Onambele-Pearson GL. The Effects of Displacing Sedentary Behavior With Two Distinct Patterns of Light Activity on Health Outcomes in Older Adults (Implications for COVID-19 Quarantine). Frontiers in Physiology. 2020;11:574595. https://dx.doi.org/10.3389/fphys.2020.574595 | Wrong population: do not reach criteria for inclusion |
| Hager A-GM, Mathieu N, Carrard S, Bridel A, Wapp C, Hilfiker R. Partially supervised exercise programmes for fall prevention improve physical performance of older people at risk of falling: a three-armed multi-centre randomised controlled trial. BMC geriatrics. 2024;24(1). doi: 10.1186/s12877-024-04927-0 | Wrong population: do not reach criteria for inclusion |
| Hsieh T-J, Su S-C, Chen C-W, Kang Y-W, Hu M-H, Hsu L-L, et al. Individualized home-based exercise and nutrition interventions improve frailty in older adults: a randomized controlled trial. The international journal of behavioral nutrition and physical activity. 2019;16(1). doi: 10.1186/s12966-019-0855-9 | Wrong population: do not reach criteria for inclusion |
| Jeon YH, Simpson J, Fethney J, Krein L, Shin M, Low LF, et al. Effectiveness of the Interdisciplinary Home-bAsed Reablement Programme (I-HARP) on improving functional independence of people living with dementia: a multicentre, pragmatic, randomised, open-label, controlled trial. Journal of Neurology, Neurosurgery and Psychiatry. 2025;96(7):705–15. https://dx.doi.org/10.1136/jnnp-2024-334514 | Wrong population: do not reach criteria for inclusion |
| Jeon Y-H, Simpson JM, Low L-F, Woods R, Norman R, Mowszowski L, et al. A pragmatic randomised controlled trial (RCT) and realist evaluation of the interdisciplinary home-bAsed Reablement program (I-HARP) for improving functional independence of community dwelling older people with dementia: an effectiveness-implementation hybrid design. BMC geriatrics. 2019;19(1). doi: 10.1186/s12877-019-1216-x | Wrong population: do not reach criteria for inclusion |
| Kyrdalen IL, Moen K, Røysland AS, Helbostad JL. The Otago Exercise Program Performed as Group Training Versus Home Training in Fall-prone Older People: A Randomized Controlled Trial. Physiotherapy Research International. 2014;19(2):108–16. 10.1002/pri.1571 | Wrong population: do not reach criteria for inclusion |
| Lytras D, Sykaras E, Iakovidis P, Komisopoulos C, Chasapis G, Mouratidou C. Effects of a modified Otago exercise program delivered through outpatient physical therapy to community-dwelling older adult fallers in Greece during the COVID-19 pandemic: a controlled, randomized, multicenter trial. European geriatric medicine. 2022;13(4):893–906. doi: 10.1007/s41999-022-00656-y | Wrong population: do not reach criteria for inclusion |
| Mousavi SA, Hin LP, Dadvar L, Hakim MN, Hamid TA, Dadgari A, et al. Randomized control trials on otago exercise program (OEP) to reduce falls among elderly community dwellers in Shahroud, Iran. Iranian red crescent medical journal. 2016;18(5):1–8. doi: 10.5812/ircmj.26340 | Wrong population: do not reach criteria for inclusion |
| Qiao X, Ji L, Jin Y, Si H, Bian Y, Wang W, et al. Effectiveness of a theory-underpinning exercise intervention among community-dwelling (pre)frail older adults: A stepped-wedge cluster-randomized trial. International Journal of Nursing Studies. 2025;161. https://dx.doi.org/10.1016/j.ijnurstu.2024.104933 | Wrong population: do not reach criteria for inclusion |
| Roberts S, Awick E, Fanning JT, Ehlers D, Motl RW, McAuley E. Long-Term Maintenance of Physical Function in Older Adults Following a DVD-Delivered Exercise Intervention. Journal of Aging and Physical Activity. 2017;25(1):27-31. https://dx.doi.org/10.1123/japa.2015-0284 | Wrong population: do not reach criteria for inclusion |
| Sharahi MY, Raeisi Z. Effects of otago and fit-and-fall proof home-based exercises on older adults’ balance, quality of life, and fear of falling: a randomized, single-blind clinical trial. Sport sciences for health. 2025;21(2):1177–86. doi: 10.1007/s11332-025-01357-2 | Wrong population: do not reach criteria for inclusion |
| Stasi S, Tsekoura M, Gliatis J, Sakellari V. Motor Control and Ergonomic Intervention Home-Based Program: A Pilot Trial Performed in the Framework of the Motor Control Home Ergonomics Elderlies’ Prevention of Falls (McHeELP) Project. Curēus (Palo Alto, CA). 2021;13(4):e14336. doi: 10.7759/cureus.14336 | Wrong population: do not reach criteria for inclusion |
| Stasi S, Tsekoura M, Gliatis J, Sakellari V. The Effects of a Home-Based Combined Motor Control and Ergonomic Program on Functional Ability and Fear of Falling: A Randomized Controlled Trial. Curēus (Palo Alto, CA). 2021;13(9):e18330. doi: 10.7759/cureus.18330 | Wrong population: do not reach criteria for inclusion |
| Suijker JJ, van Rijn M, Buurman BM, ter Riet G, Moll van Charante EP, de Rooij SE. Effects of Nurse-Led Multifactorial Care to Prevent Disability in Community-Living Older People: Cluster Randomized Trial. PloS one. 2016;11(7):e0158714. https://dx.doi.org/10.1371/journal.pone.0158714 | Wrong population: do not reach criteria for inclusion |
| Suikkanen S, Soukkio P, Aartolahti E, Kääriä S, Kautiainen H, Hupli MT, et al. Effect of 12-Month Supervised, Home-Based Physical Exercise on Functioning Among Persons With Signs of Frailty: A Randomized Controlled Trial. Archives of Physical Medicine and Rehabilitation. 2021;102(12):2283–90. https://dx.doi.org/10.1016/j.apmr.2021.06.017 | Wrong population: do not reach criteria for inclusion |
| Tuvemo Johnson S, Anens E, Johansson AC, Hellström K. The Otago Exercise Program With or Without Motivational Interviewing for Community-Dwelling Older Adults: A 12-Month Follow-Up of a Randomized, Controlled Trial. Journal of Applied Gerontology. 2021;40(3):289–99. https://dx.doi.org/10.1177/0733464820902652 | Wrong population: do not reach criteria for inclusion |
| van Het Reve E, Silveira P, Daniel F, Casati F, de Bruin ED. Tablet-Based Strength-Balance Training to Motivate and Improve Adherence to Exercise in Independently Living Older People: Part 2 of a Phase II Preclinical Exploratory Trial. Journal of Medical Internet Research. 2014;16(6):e159 https://dx.doi.org/10.2196/jmir.3055 | Wrong population: do not reach criteria for inclusion |
| Vittala G, Sundari L, Basuki N, Kuswardhani R, Purnawati S, Muliarta I. The addition of active stretching to balance strategy exercise is the most effective as a home-based exercise program in improving the balance of the elderly. Journal of mid-life health. 2021;12(4):294–8. doi: 10.4103/jmh.jmh_184_21 | Wrong population: do not reach criteria for inclusion |
| Vogler CM, Sherrington C, Ogle SJ, Lord SR. Reducing Risk of Falling in Older People Discharged From Hospital: A Randomized Controlled Trial Comparing Seated Exercises, Weight-Bearing Exercises, and Social Visits. Archives of Physical Medicine and Rehabilitation. 2009;90(8):1317–24. https://dx.doi.org/10.1016/j.apmr.2009.01.030 | Wrong population: do not reach criteria for inclusion |
| Walters K, Frost R, Avgerinou C, Kalwarowsky S, Goodman C, Clegg A, et al. Clinical and cost-effectiveness of a home-based health promotion intervention for older people with mild frailty in England: a multicentre, parallel-group, randomised controlled trial. The Lancet Healthy longevity. 2025;6(2). https://dx.doi.org/10.1016/j.lanhl.2024.100670 | Wrong population: do not reach criteria for inclusion |
| Wongcharoen S, Sungkarat S, Munkhetvit P, Lugade V, Silsupadol P. Home-based interventions improve trained, but not novel, dual-task balance performance in older adults: A randomized controlled trial. Gait & posture. 2017;52(NA):147–52. doi: 10.1016/j.gaitpost.2016.11.036 | Wrong population: do not reach criteria for inclusion |
| Wood L, Comer C, Newell P, Ribeiro DC, Williamson E, Lamb SE. Exploring the mediators of the BOOST intervention on walking disability at 12 months: a causal mediation analysis. Journal of physiotherapy. 2025;71(3):185–91. doi: 10.1016/j.jphys.2025.05.012 | Wrong population: do not reach criteria for inclusion |
| Yerlikaya T, Öniz A, Özgùren M. The effect of an interactive tele rehabilitation program on balance in older individuals. Neurological sciences and neurophysiology. 2021;38(3):180–6. doi: 10.4103/nsn.nsn_91_21 | Wrong population: do not reach criteria for inclusion |
| Bårdstu HB, Andersen V, Fimland MS, Aasdahl L, Raastad T, Cumming KT, et al. Correction to: Effectiveness of a resistance training program on physical function, muscle strength, and body composition in community-dwelling older adults receiving home care: a cluster-randomized controlled trial. European Review of Aging and Physical Activity. 2020;17(1). https://dx.doi.org/10.1186/s11556-020-00245-7 | Wrong publication type |
| Bighea A, Patru S, Bumbea A, Popescu R. Randomized controlled trial of a home based exercise and balance training programme in elderly women with osteoporosis. *Osteoporosis International.* 2011;22:238-8. | Wrong publication type |
| Dunlop R. An inexpensive and accessible exercise regime significantly improves balance and reduces injuries in the elderly. Focus on alternative and complementary therapies. 2011;16(1):56–7. doi: 10.1111/j.2042-7166.2010.01070_11.x | Wrong publication type |
| Pyatak EA, Carlson M, Vigen CLP, Blanchard J, Niemiec SS, Sideris J, et al. Contextualizing the Positive Effects of the Well Elderly 2 Trial: A Response to Schelly and Ohl (2019). The American Journal of Occupational Therapy. 2019;73(6):1-11. 10.5014/ajot.2019.038752 | Wrong publication type |
| Stark S, Keglovits M, Somerville E. A Randomized Controlled Feasibility Trial of Tailored Home Modifications To Improve Activities of Daily Living. The American Journal of Occupational Therapy. 2016;70(4_Supplement_1):7011520290-7011520290p1. 10.5014/ajot.2016.70S1-RP103E | Wrong publication type |
| Szanton SL, Leff B, Li Q, Breysse J, Spoelstra S, Kell J, et al. CAPABLE program improves disability in multiple randomized trials. Journal of the American Geriatrics Society (JAGS). 2021;69(12):3631–40. 10.1111/jgs.17383 | Wrong publication type |
| Edwards M. Hospital and home rehabilitation did not differ for functional competence in activities of daily living. Evidence Based Nursing. 2009;12(3):84. 10.1136/ebn.12.3.84 | Wrong publication type: Commentary |
| Frost R, Avgerinou C, Kalwarowsky S, Mahmood F, Goodman C, Clegg A, et al. Enabling health and maintaining independence for older people at home (HomeHealth trial): a multicentre randomised controlled trial. The Lancet (British edition). 2023;402:S42–S42. https://dx.doi.org/10.1016/S0140-6736(23)02071-8 | Wrong publication type: Meeting Abstracts |
| Crotty M, Giles LC, Halbert J, Harding J, Miller M. Home versus day rehabilitation: a randomised controlled trial. Age and ageing. 2008;37(6):628–33. https://dx.doi.org/10.1093/ageing/afn141 | Wrong setting: at hospital |
| Lewin G, Clemson L, Boldy D, Burton E. Long-term Benefits of a Lifestyle Exercise Program for Older People Receiving a Restorative Home Care Service: A Pragmatic Randomized Controlled Trial. Healthy Aging ＆ Clinical Care in the Elderly. 2014;2014(2014):1–9. doi: 10.4137/HACCE.S13445 | Wrong study design |
| Watanabe R, Kojima M, Yasuoka M, Kimura C, Kamiji K, Otani T, et al. Home-Based Frailty Prevention Program for Older Women Participants of Kayoi-No-Ba during the COVID-19 Pandemic: A Feasibility Study. International journal of environmental research and public health. 2022;19(11):6609. doi: 10.3390/ijerph19116609 | Wrong study design |
| Garbin AJ, Tran MK, Graber J, Derlein D, Currier D, Altic R, et al. Improving Function in Older Adults With Hospital-Associated Deconditioning: Lessons Learned Comparing a Randomized Controlled Trial to Real World Practice. Physical Therapy. 2024;104(12). https://dx.doi.org/10.1093/ptj/pzae173 | Wrong study design: non-randomized control group |
| Takatori K, Matsumoto D, Nishida M, Matsushita S, Noda T, Imamura T. Benefits of a novel concept of home-based exercise with the aim of preventing aspiration pneumonia and falls in frail older women: a pragmatic controlled trial. BMJ Open Sport & Exercise Medicine. 2016;2:e000127. https://dx.doi.org/10.1136/bmjsem-2016-000127 | Wrong study design: non-randomized controlled trial |
| Gitlin LN, Winter L, Dennis MP, Hauck WW. Variation in Response to a Home Intervention to Support Daily Function by Age, Race, Sex, and Education. The Journals of Gerontology Series A, Biological Sciences and Medical Sciences. 2008;63(7):745–50. https://dx.doi.org/10.1093/gerona/63.7.745 | Wrong study design: subgroup analysis |
