# Supplementary material for: The components and effects of home rehabilitation on activities of daily living and physical performance of community dwelling older people with low physical performance – a systematic review and meta-analysis of randomized controlled trials
Source: BMC Geriatr. 2026 Jun 30;26:889. doi: 10.1186/s12877-026-07887-9 (PMC13321581; doi:10.1186/s12877-026-07887-9)
Supplement: Supplementary file 4 — Additional file 4. Key characteristics of the included studies. [file 12877_2026_7887_MOESM4_ESM.docx]

**Additional File 4.** Key characteristics of the included studies.

| **Intervention** | **Study** | **Purpose** | **Population** | **Intervention** | **Control** |
| --- | --- | --- | --- | --- | --- |
| **Activity-based** | de Vriendt, 2016,^47^  Belgium | Client-centered and activity-oriented  intervention program effectiveness on BADL and HRQL | n=168  Age IG M 79.9  Female IG 76%  >1 BADL functional limitation | **Interventionist:** OT  **Purpose of the intervention:** Secondary prevention of disability  **Components:** Client-centered goal-setting (self-care, productivity, leisure activities), Comprehensive Geriatric Assessment, open interviewing for clear understanding functional problem and impact on HRQL; Negotiating therapy plan; Treatment phase (training skills and functions (cognitive, sensory-motor)), educate in-/formal caregiver, advise and instruct assistive product use, comprehensive intervention); Evaluation, report to community care and physician  **Staff preparation:** Course intervention protocol  **Dosage:** 8-10 wks, 1-3 visits  **Adherence:** Mean time 134 min, 33% >3 visits, n=4 declined visits | Usual care, i.e. community care services support in housekeeping, self-care; nursing healthcare support; social worker follow-up |
|  | Friedman, 2009,^48^ USA | Disease self-management health promotion effect on patient and informal caregivers’ satisfaction, patient empowerment,  health and disability status | n=766  Age M 77.3  Female 68.8%  Dependent 2 BADL or 3 IADL or more | **Interventionist:** RN  **Purpose of the intervention:** Improved health and disability status  **Components:** Empowering disease self-management health promotion; Assess medication, vital signs; Negotiated/individual goals; Health education planning (prevent, promote, chronic disease self-care, health behavior change maintenance); Physical activity components; Communication facilitating conference (RN, physician, patient, family); Self-care handbooks in collaboration with RN  **Staff preparation:** Fitness specialist training  **Dosage:** 24 mo (mean 579 d). 19 monthly visits  **Adherence:** 67.3% completed 24 mo demonstration  **Both groups:** Medicare beneficiaries | Not reported |
|  | Gitlin, 2006,^28^ USA | Multicomponent intervention efficacy on functional difficulties, fear of falling, home hazards, enhance self-efficacy, adaptive coping | n=319  Age M 79  Female 82%  Difficulty or need help ≥1 BADL or 2 IADL | **Interventionist:** OT, PT  **Purpose of the intervention:** Reduce difficulties in performing everyday tasks  **Components:** Problem solving to identify behavioral and environmental contributors to performance difficulties; Strategy use; Muscle strength and balance exercise, fall-recovery techniques; Home modification; Equipment provision; Future needs adjustments  **Staff preparation**: 35h training (reading and guided practice clinical interviewing) before study and supervision meetings throughout the study.  **Dosage:** 6 mo. Including 5x90 min home visits (OT, PT), 1 phone call, 3 follow-up phone calls, 1 final home visit  **Adherence:** Not reported | No intervention |
|  | Hagelskjær, 2023,^29^ Denmark | Individualized OT intervention program Activity-Based Lesson for Everyday life  (ABLE) effectiveness on ADL | n=78  Age IG MD 75  Female IG 76%  Experience ADL problems and motivated | **Interventionist:** OT  **Purpose of the intervention:** Enhancing ADL ability  **Components:** ADL assessment (interview, observation); Goal setting; Individualized intervention components (habits, attitude, prioritize), physical/social environment, tools, services/opportunities, minor steps, simplify); Adaptation approach (environment, assistive products, routines, consultation, education)  **Staff preparation**: 3.5-day course ABLE 2.0 program  **Dosage:** 2 mo. Including 5x 1h home visits (max 8)  **Adherence:** Delivered through home visits 94.4%, phone 5.6%. | Usual municipality OT specific ADL tasks  **Dosage:** 2 wks, including 2x 1h home visits  **Adherence:** delivered through home visits 94.7%, phone 5.3%  M 14.5 d |
|  | Nielsen, 2019,^30^ Denmark | Intensive client-centered OT (ICC-OT) effect on self-rated occupational performance | n=119  Age IG M 78  Female IG 76%  Experience occupational performance problems, apply for/receive home care services | **Interventionist:** OT  **Purpose of the intervention:** Improve occupational performance  **Components:** Client-centered ADL assessment; Personalized goals (home/local community) collaboratively addressing the person’s preferences;  Individual tailored built on acquisitional, adaptive, and restorative models; Practicing tasks/activities to reach goals  **Staff preparation**: 5h x2 workshops before and follow-ups throughout the intervention  **Dosage:** 3 mo (first week baseline assessment), home visit 2/wk  **Adherence:** On average 11h ICC-OT (n=52); 5.4h PT (n=9); Assistive product 39 (n=24)  **Both groups:** Municipality home care services (personal care, practical help, meal delivery, home care reablement, OT, PT, minor home modifications, assistive products) | Usual care possible referral to reablement specific BADL, household tasks. Including home visits by home care assistants, possible 3 home visits by municipality OT  **Dosage:** up to 3 wks  **Adherence**: On average 3.1h OT (n=14); 6.6h PT (n=5); Assistive product 15 (n=9) |
|  | Sheffield, 2013,^31^ USA | Evaluate restorative OT to usual care | n=71  Age M 82  Female 80%  Receiving/on waiting list for agency services | **Interventionist:** OT  **Purpose of the intervention:** Balance safety and independence during daily activities  **Components:** In-home assessment of daily activities; Client–family collaboration mutual goals; Home modifications; Provision/training assistive product; Medication management; Education in adaptive and compensatory strategies to improve safety and independence  **Staff preparation**: 2-day training of program, videotapes and mentorship before study and supervision throughout the study  **Dosage:** 3 mo. Including 4 home visits  **Adherence:** Not reported | Usual care (no OT)  a) Waiting list: case manager contact  b) Receiving services: in-home aid services, medical supplies, emergency responses, case manager contact |
|  | Stark, 2018,^49^ USA | Home modification efficacy on risk of falls | n=92  Age IG 78.4  Female IG 78%  Impairment ADL and iADL (screening Older Americans Resources and Services Multidimensional Functional Assessment Questionnaire) | **Interventionist:** OT, OT assistants  **Purpose of the intervention:** Process evaluation follow-up of compensating functional ADL limitations by tailored home environmental modification  **Components:** Performance-based assessment of abilities and environment, ten most problematic activities, photograph barriers; Shared decision-making tool; Coordinate installation; Training modification and equipment safely for independence; Problem solve of remaining issues  **Staff preparation:** 15h training (self-reading, lecture, case studies, mentored learning)  **Dosage:** 60 d, 6x 90-min visits, total 540 min  **Adherence:** 5.9 visits, 84.39 min, 91.24 d (total 496.43 min), over 90% delivery accuracy, 91% adherence at 12 mo | OT visits, standardized kit adaptive equipment fine motor tasks (needle threader, large-grip pizza cutter)  **Dosage:**  60 d, 6x 90-min visits, total 540 min  (equal time from OT) |
|  | Szanton, 2011,^33^ USA | Program Community Aging in Place Advancing Better Living for Elders acceptability, feasibility, effect on disability | n=40  Age M 78  Female 95%  Difficulty with ≥1 BADL or 2 IADL | **Interventionist:** OT, RN  **Purpose of the intervention:** Reduce disability  **Components:** Multidisciplinary assessment (OT: ADL safety, efficiency, difficulty, barriers/supports, home safety; RN: pain, depression, medication, contact primary care, muscle strength and balance); Integrated plan (collaboratively identify goals and tailored strategies, tools as Motivational Interviewing); Strategies (energy-conserving, simplifications, balance/fall-recovery techniques, mood and pain management, muscle strength and balance exercise, Tai Chi); Home repair, environmental modifications, medical equipment/assistive product, general approach to address other daily situations  **Staff preparation:** Not described  **Dosage:** 6 mo. Including 10x1h-home visits distributed across wks for supervised sessions followed by unsupervised practice  **Adherence:** Not reported | Supervised sedentary activity of choice with research assistant (not OT, RN)  **Dosage**: up to 10x1h sessions  **Adherence:** Not reported |
|  | Szanton, 2019,^34^ USA | Program Community Aging in Place Advancing Better  Living for Elders effect on disability | n=300  Age M 76  Female IG 88%  Low income  Difficulty with ≥1 BADL or 2 IADL | **Interventionist:** OT, RN  **Purpose of the intervention:** Reduce disability  **Components:** Multidisciplinary assessment (OT: functional disability, home safety risks, functional goals; RN: goal pain level, depression, medication, contact primary care, muscle strength and balance); Integrated plan (collaboratively tailored strategies, tools as Motivational Interviewing); Strategies (energy-conserving, simplifications, balance/fall-recovery techniques, behavioral activation for depressive symptoms and balance issues, pain management, muscle strength and balance exercise); Home repair, environmental modifications, medical equipment/assistive product, education address future needs  **Staff preparation:** Not described  **Dosage:** 5 mo. Including 10x1h-home visits spaced across wks for supervised sessions followed by unsupervised practice  **Adherence:** 9.1 visits (92.8% 8 of 10 sessions, 3.9% less than 3 sessions) | Supervised sedentary activity of choice (not OT, RN)  **Dosage:** up to 10 1h sessions with research assistant  **Adherence:** 49.3% 8 of 10 sessions, 37.8% less than 3 sessions |
|  | Szanton, 2025,^50^ USA | Program Community Aging in Place Advancing Better  Living for Elders effect on disability | n=268  Age M 75  Female 72%  Difficulty with ≥1 ADL after home care episode post hospital discharge | **Interventionist:** OT, RN  **Purpose of the intervention:** Reduce disability  **Components:** Multidisciplinary assessment (OT: functional disability, home safety risks, functional goals; RN: goal pain level, depression, medication, contact primary care, muscle strength and balance); Integrated plan (collaboratively tailored strategies, tools as Motivational Interviewing); Strategies (energy-conserving, simplifications, balance/fall-recovery techniques, behavioral activation for depressive symptoms and balance issues, pain management, muscle strength and balance exercise); Home repair, environmental modifications, medical equipment/assistive product, education address future needs  **Staff preparation:** Not described  **Dosage:** 5 mo. Including 10x1h-home visits spaced across wks for supervised sessions followed by unsupervised practice  **Adherence:** 74.6% 8 to 10 visits | No intervention |
|  | Whitehead, 2016,^36^ England | Feasibility RCT: OT ADL intervention added to home care reablement services | n=30  Age IG M 83  Female IG 27%  Users of home care reablement services | **Interventionist:** OT  **Purpose of the intervention:** Maximize independence in B/IADL through OT skills (principles, practices, process).  **Components:** OT combined medical knowledge of prognosis with functional ability assessment to select intervention approach. ADL program individually tailored and agreed with participant (goal setting; practicing activities; graded process re-learning, building skills to manage ADL independently; adapt activity, environmental modification; equipment provision; case management involving advice to person and their support network).  **Staff preparation:** OT continuous contact with team (PT, RN, chiropodist) and services as appropriate  **Intervention Delivery:** Goal assessment, activity practice, weekly review (OT)  **Dosage:** Average 8 wks (up to 4.5 mo). Including average 5x 45-min home visits  **Both groups:** Routine home care reablement services  **Adherence:** Home visit time included 29% assessment, 24% support (advice, administration), 19% training, 12% goal reviewing, 11% teaching, 5% goal setting | Usual care home care reablement service (social care workers and managers, no qualified health professionals)  **Dosage:** 6 wks |
|  |  |  |  |  |  |
| **Exercise-based intervention** | Bjerk, 2019,^51^ Norway | Falls prevention exercise  effects on HRQL, physical function, falls self-efficacy | n=155  Age M 82.7  Female 79.3%  Receive home care | **Interventionist:** PT  **Purpose of the intervention:** Falls prevention  **Components:** Individually adjusted exercise program (strengthening lower body, balance (standing, walking backwards, stairs, chair rising); Information exercise safety, fall prevention, ADL; Ankle cuffs (1-2,5kg), exercise manual, exercise log  **Staff preparation**: 1 day workshop fall prevention Otago Exercise Program. ½-day fidelity workshop every 4^th^ month  **Dosage:** 12 wks. Including 3/wk 30 min-exercise, increased repetitions and weights, 2/wk 30 min-walk if safe. Weekly supervision face-to-face or motivational phone calls, 5x 1h-home visits (wk 1, 2, 4, 8, 10), 4 additionally if required  **Adherence:** 73.5% prescribed (visits, calls, exercise) | Usual primary healthcare service (referral to community health services when required (e.g. malnutrition)) |
|  | Boongird, 2017,^26^ Thailand | Primary care fall prevention program effect on falls, improve strength, balance, fear of falling, HRQL | n=439  Age IG M 74  Female IG 84%  Mild/moderate balance dysfunction | **Interventionist:** RN, monthly team meeting (RN, physician, researchers) device, extra training, health problems  **Purpose of the intervention:** Improve muscle strength, balance, fear of falling, number of falls, quality of life  **Components:** 5 simple progressed (modified Otago Exercise Program) lower body exercises strength, stretch, balance, walking; 1h training instruction; Video, manuals, exercise log  **Staff preparation:** Not described  **Dosage:** 12 mo. Including 6/wk 20 min exercise, 8RM (to 10RM, fatigue up to 3 more), 2/wk 30 min walking. 1 home visit, regular weekly phone calls decreased overtime  **Adherence:** Exercised ≥120 min/wk increased overtime from 29.6% to 56.8%  **Both groups:** Fall prevention education: video, books home safety and fall prevention | Fall prevention education |
|  | Burton, 2013,^52^ Australia | Lifestyle functional exerciser program effect on short-term restorative home care service program for independence after discharged from hospital or simply in need of short-term assistance | n=80  Age IG M 80  Female IG 75%  Medium to high level of dependency IG 84% | **Interventionist:** PT, OT, RN  **Purpose of the intervention:** Improve balance, muscle strength, fall prevention by embedding exercise into everyday activities  **Components:** 7 balance exercises (standing, walking), 6 lower body strength exercises (from ankle rotations to stairs) with examples of challenging exercises; Exercise manual, exercise log  **Staff preparation:** Training session  **Dosage:** 8 wks (for hospital discharge 10 wks). Including every day incorporated in daily tasks/routines. 3 home visits every other week  **Adherence:** Exercised 4.91/w, three quarters completed daily exercise log  **Both groups:** Interdisciplinary restorative  home care services (care manager OT, PT, RN, assisted by aides where required) | PT, OT, RN  Usual care including structured exercise program (modified Otago Exercise Program) lower body exercises strength, balance, exercise manual, exercise log  **Dosage:** 8 wks. Including 3/d 15-20 min exercise,  5 repetitions. Progressed to level 2, 5 repetitions. 3 home visits  **Adherence:** Exercised 4.42/w, two thirds completed daily exercise log |
|  | Cederbom, 2019,^27^ Norway | Individually tailored integrated behavioral medicine approach in PT (BMPI) effects on pain-related disability, physical function, pain, pain-related beliefs, physical activity, falls efficacy, HRQL | n=105  Age M 85  Female 88%  Dependent on in/formal care ≥1/m B/IADL | **Interventionist:** PT  **Purpose of the intervention:** Reach the participants own mobility goal in daily living  **Components:** Functional Behavioral Analysis to identify goal behavior; Functional progressed exercises; Training basic physical and psychological skills (self-efficacy, decreasing catastrophizing thoughts, fear of falling and movement); Training skills to achieve goal behavior and more complex skills for other behaviors, secondary activity goals  **Staff preparation:** theoretical framework education, training in intervention delivery  **Dosage:** 10 wks. Including 9 continuous home visits. Dosage based on ability to sustain/increase progressively, considering function and health status  **Adherence:** Exercised >5/wk 32% (mean 4 times/wk), >3/wk 89%; training goal behavior 1/wk 80% (mean 3/wk)  **Both groups**: General advice (oral, written) on physical activity for older people having chronic musculoskeletal pain | Usual care (advice and information on official recommendations on physical activity not physical exercise)  **Dosage:** 10 wks. Including 9 phone calls |
|  | Clegg, 2014,^53^ United Kingdom | Feasibility of exercise program to improve mobility and function in frail older people | n=84  Age M 79  Female 71%  Housebound (currently case managed, day center, respite care, assisted living, hospital discharged) | **Interventionist:** PT  **Purpose** **of the intervention:** Improve muscle strength, mobility, balance, aerobic capacity, functional mobility (bed, chair, toilet, short walking distance)  **Components:** Progressive 3-level strengthening exercise program; Behavioral change techniques (information value exercise, specific functional goals, face-to-face and paper instructions, weekly encouragement, gentle exercises for worse d, self-log, agreed progression, fridge magnet reminder); Exercise manual (information, safety tips, good posture, exercise, staying on track)  **Staff preparation**: 2h-workshop intervention training  **Dosage:** 12 wks. Including 3/d for 5/wk 15 min-exercise. Progression up to 15 repetitions wk 4, new exercises or new program level. 5 home visits, 7 phone calls  **Adherence:** completion 64% | Usual care primary healthcare team |
|  | Courtney, 2012,^54^ Australia | Multifaceted  transitional care intervention including hospital and  home-based exercise strategies effectiveness on functional status and ADL independence | n=128  Age M 78  Female 62%  Functional impairment/ depression history | **Interventionist:** RN  **Purpose of the intervention:** Improve health-promoting behaviors. strength, stability, endurance, mobility  **Components:** Individual tailored enhancing exercise program strengthening upper and lower body, balance, stretch, walking (aerobic, mobility); Home modification; Address transitional concerns within 48h-discharge; Continuous feedback treatment regime management, health promotion, exercise plan, goal adjustment, adherence  **Staff preparation:** Not described  **Dosage:** 24 wks. Including muscle strength 3-4/wk 2-3x10; daily balance training; slow to moderate walking 10-15 min 3-4/wk. Weekly phone calls first month, monthly following 5 mo. 1 home visit  **Adherence:** 50-60% adherence “all or most of the time”, 20% “some of the time”, 24-36% “none or little”. 40-50% all 4 exercise components, muscle strength component least used, 30-40% only walking  **Both groups**: Usual care | Usual care discharge planning, follow-up care, rehabilitation advice |
|  | Garbin, 2024,^55^ USA | High-intensity progressive, multi-component (PMC) effect on physical function after hospital or skilled nursing facility discharge | n=200  Age M 78.1  Female 58%  Referral home PT acute medical deconditioned from hospitalization | **Interventionist:** PT  **Purpose of the intervention:** Combining high-intensity exercise, enhanced care transition, protein supplementation  **Components:**  Progressive strengthening lower body, equipment (6-100 pounds leg press, hip extension); Progressed ADL training (transfer); Progressed gait training (step/walking complexity, speed); Home exercise program (chair rise, step-up, walking); High-protein 15g/d (post training); Care transitions coordinator contact (RN or PT) patient-centered, interdisciplinary care plan including visit 1-3h; Exercise and nutrition log  **Staff preparation:** PMC training  **Dosage:** 60 d. Including muscle strength 3x8 8RM, reevaluated weekly, home exercise 2/d (ADL up to 8 repetitions, walking 20 min). 12 home visits, twice weekly first month, 1/wk last month  **Adherence:** Muscle strength 95.4%, ADL 90.1%, gait 83.8%. 10.1 of 12 visits  **Both groups**: Initiation within 5d post discharge. Agency standard home healthcare (RN, OT, social work visits as needed) | Enhanced usual Care (more PT visits than clinic, progression standardized), no care transitions coordinator contact, nutritional advice or supplementation.  Interventionist: PT  **Components:** Strengthening (active range of motion, unweighted).  Safe, upper body supported ADL training (transfers).  Safe gait training (tailored inside/outside).  Home exercise program 2 exercises 10 repetitions/d  **Staff preparation:** enhanced UC training  **Dosage:** Equal frequency, duration, domain intervention, 1x 10 repetitions, from 4 up to max 7 exercises progression  Adherence: Muscle strength 97.3%, ADL 67.4%, gait 94.8%. 10.2 of 12 visits |
|  | Sherrington, 2014,^56^ Australia | Home-based exercise program effects on falls and mobility after recently hospital discharge | n=340  Age M IG 82  Female IG 72%  Baseline assessment detected physical performance limitations | **Interventionist:** PT  **Purpose of the intervention:** Enhance balance, mobility, prevent falls by exercises similar to daily activities.  **Components:** Progressive lower body exercises strength, balance (modified Weight-bearing Exercise for Better Balance program), equipment weight-belt/vest, small step-up blocks; Encourage self-training through Physical Activity Stage of Change model to encourage self-training; Exercise manual, Exercise log  **Staff preparation:** Not described  **Dosage:** 12 mo. Individually tailored level of difficulty and number of repetitions, 6/w 20-30 min exercise. 10 home visits, more frequently in the beginning for safety, tailoring, progression  **Adherence:** Completed exercise sessions decreased from MD 5 to 3/w and completed exercises from 77% to 47%. 9.2 home visits  **Both groups**: Fall prevention information booklet | Fall prevention information booklet |
|  | Siemonsma, 2018,^57^ Netherlands | Functional task exercise (FTE) effects on daily functioning | n=155  Age IG MD 84  Female IG 72%  Daily activity limitations (predicting ISCOPE screening 2 or more positive answers) | **Interventionist:** PT  **Purpose of the intervention:** Prevent age-related functional decline  **Components:**  Functional diagnostics in domain of daily functioning; Mutual decision of daily activity training for independence; Engage social support; Exercises linking cognitive, perception, execution of task; Problem-orientated, situational and task-specific to build confidence; Progressed physical, perceptual, cognitive; Four movement domains (lying to standing, walking, stairs, transport object) from 2 domains, 3x 5-10, tailored complexity (3 phases: 2w learn exercise, 4w irregular floors, pick up from floor, distract - count backwards, 6w all domains matching individual daily activities); Engaged social environment in training stimulating performance of daily activities; Motivating and advising being daily physically active  **Staff preparation:** 44h FTE training education  **Dosage:** 3 mo. Including max 18 30-min sessions, at least 2 of four domains 3x5-10  **Adherence:** Not reported  **Both groups:** One clinic included PTs from IG and CG | Preventive PT (PPT)  Regular PT decide intervention and location, open referral help daily functioning, following national PT guidelines  **Dosage:** 3 mo, up to 18 30-min sessions  **Adherence:** Not reported  Not included in this review: Control group from other study (ISCOPE) |
|  | Stevens-Lapsley, 2016,^58^ USA | High-intensity, progressive multicomponent exercise feasibility and effective on functional mobility | n=22  Age IG M 87  Female IG 58.3%  Homebound, risk functional decline, referral to home PT after acute hospitalization | **Interventionist:** PT  **Purpose of the intervention:** Reduce adverse health events  **Components:** Progressive multicomponent strengthening lower and upper body; ADL; Home exercise lower and upper body; Weekly reassessment; Body weight, progressed resistance equipment 2.72-45.36 kg (supine leg press, hip ext, plantar flexion, seated press and row)  **Staff preparation:** one PT trained in progression  **Dosage:** 1 mo. Including home visits 2-3/wk strengthening training 2-3x8 (8RM). ADL accomplish transfer indoors then car transfer, dual task. Gait 10- 20-min 2/d.  **Adherence:** PT visits 9.67, duration 30.58 d | PT  Usual care community standard home PT  (low intensity strengthening exercise, simple home ambulatory skills, basic in-home functional mobility training, low intensity home exercise program)  **Dosage:** 1 mo. 2-3 visits/w  **Adherence:** PT visits 8, duration 34.8 d |
|  | Stevens-Lapsley, 2023,^32^ USA | Progressive, high-intensity resistance training affects physical function and its safety | n=150  Age IG M 77  Female IG 23%  Veterans and spouses  Referred for home health PT with deconditioning due to acute hospitalization/ COVID stay-at-home policies | **Interventionist:** PT  **Purpose of the intervention:** Improve physical function  **Components:** Progressive high-intensity resistance training lower body; Functional training ADL (transfer) patient-specific progressive; Patient specific motor control gait training; Unsupervised body-weight exercise, walking, exercise log  **Staff preparation:** Trained to deliver PHIT  **Dosage:** 30 d. Including home visits 3/wk with strengthening training 3x8 (8RM), ADL (5-10 min) 8x1 to fatigue, gait 10 min x1 to fatigue. Unsupervised days 3 body-weight exercise, 10- or 20-min walking bouts.  **Adherence:** Strengthening training, ADL training completed in >90% of sessions, motor control training in >80% of sessions | Standard PT  Strength, functional, and gait training, exercise log  **Dosage:** 30 d. Including home visits 3/wk unweighted, 2/wk unsupervised  **Adherence:** Exercises completed in >90%, ADL completed in 55% of sessions |
|  | Whitney, 2019,^59^ USA | Tailored and progressed exercise program Zōntago added to fall preventive program Safe Strides effect on ADL | n=112  Age M 82  Female IG 60%  Home bound, ADL dependent (13.5 (3.5) Outcome and Assessment Information Set (OASIS), low score indicates improvement (0-30)) | **Interventionist:** PT, PT assistants  **Purpose of the intervention:** Motivate individualized physical exercise adherence  **Components:** Motivational interviewing (positive health behavior - identification, prioritization, reinforcement); Tailored progressive strengthening lower body, balance, walking (modified Otago Exercise Program); Dose-specific (mod Borg); Task-specific (chair rise); Exercise log  **Staff preparation:** 19.5h Safe Strides training, 8h Zōntago training and 3 follow-up labs/sessions  Intervention delivery:)  **Dosage:** 56 d duration, 30-min exercise program 3/wk, walking program 2/wk. Moderately challenge 70% of session or more (13-17 RPE, 3-4 RPD), progressed resistance, repetitions, time, difficulty, support 28 visits  **Adherence:** 56 d, 28 visits  **Both groups:** Safe Strides particle repositioning maneuver, vestibular rehabilitation | PT  Safe Strides comprehensive and extensive training fall reduction program  **Staff preparation:** 19.5h comprehensive falls reduction e-learning, facilitated training, laboratory skills validation, post-training laboratories, in-home skills validation) |
|  | Yang, 2012,^37^ Australia | Personalized, homebased exercise program effect on mild balance dysfunction | n=165  Age IG M 81  Female IG 45%  Mild balance dysfunction/balance concerns | **Interventionist:** PT  **Purpose of the intervention:** Revers mild balance dysfunction  **Components:** Progressed personalized home exercise program (Otago Exercise Program and Visual Health Information Balance and Vestibular Exercise Kit based) (muscle strength and balance exercises lower body, tailored walking program); Ancle cuffs weights, exercise manual, exercise log; Monitored, progressed, and adherence support at home visits  **Staff preparation**: Not described  **Dosage:** 6 mo. Including 5/wk, 5-8 exercises, graduated walking program aiming for ≥30 min/day, home visit wk 1, 4, 8  **Adherence:** Exercise >5/wk 44.1%, 3-4/wk 39%, <2/wk 13.6% | Fall prevention information booklet |
|  |  |  |  |  |  |
| **Reablement-based** | Gustafsson, 2025,^60^ Sweden | Intensive home reablement (IHR) effect on overall life satisfaction, self-assessed health, HRQL, subjective well-being, physical activity capacity, homecare hours | n=237  Age IG M 83.6  Female IG 76%  Applied home service homecare receiving IHR | **Interventionist:** Interprofessional team assistant nurse, RN, PT, OT, social worker  **Purpose of the intervention:** Regain/maintain functional ability physically, mentally, socially for independent living with optimal health and wellbeing  **Components:** Trained and coordinated interdisciplinary team; Team planning with participant following overall goals accepted service (short/long term goals e.g. climb stairs/share bedroom, shower/autonomy, ride bus/see friends); High care continuity (double CG), geographical closeness of staff, follow-ups of individuals at regular interprofessional meetings  **Staff preparation**: 4-5 wks university course reablement (health perspectives, rehabilitation process (goal-setting, teamwork, motivational talks), evidence-based practice, research participation)  **Staff collaboration:** 2 contact persons/participant twice time ordinary home-based care and homecare services of nursing (personal hygiene, supervision, support medication) and social service (purchasing, cleaning, laundry). Visits of team members  **Dosage:** 3 mo. Visits up to several times/d  **Adherence:** Not reported | Usual care according to municipality’s previous prevailing homecare, rehabilitation (aid assessors and profession of relevance assistant nurse, RN, PT, OT, social worker) |
|  | Rooijackers, 2021,^61^ Netherlands | “Stay active at home” (SAaH) effectiveness  on sedentary behavior, daily functioning, physical  functioning, psychological functioning, falls | n=264  Age M 82.1  Female 67.8%  Receiving homecare services (personal 87 domestic 55.7 nursing 52.7) | **Interventionist:** Homecare staff (RN, assistant nurses, nurse aides)  **Purpose of the intervention:** Equip homecare staff self-efficacy in reablement (knowledge, attitude, skills), integrated by social and organizational support  **Components:** Reablement training program (motivating, engagement daily activities and physical activity; Goal-setting and action planning; Social network; Assess capabilities  **Staff preparation:** 2h kick-off meeting,1h bi/monthly meeting for 6 mo, 2h booster session at 9 mo (role-play with professional actors); Practical assignment discussions, interactive teaching, mentoring; Weekly newsletters; Manager invited.  **Staff collaboration:** District nurse set goals and action plans with care receiver in collaboration with team  **Dosage:** Not reported  **Adherence:** Staff training 56.6-73.4% meetings, assignments, newsletters  **Both groups:** Nursing team personal care and nursing care several visits/d. Domestic workers domestic support multiple hours once/wk. One district nurse/team supervised and coordinated | Usual care no staff training, usual care delivery |
|  | Tuntland, 2015,^35^ Norway | Reablement effectiveness on daily activities, physical functioning, HRQL | n=61  Age IG M 80  Female 69%  Functional decline ≥1 ADL | **Interventionist:** OT, PT, home-care service personnel  **Purpose of the intervention:** Enhancing performance of daily activities defined as important by the person  **Components:** Assessment (OT/PT); Rehabilitation plan personalized goals; Home-care service supervised to stimulate self-performed ADL, assist daily training; Adapt activity, environment modifications; Exercise program  **Staff preparation:** Training including self-management ideology (OT, PT, home-care service), assessments (OT, PT). Weekly informal meetings with home-care service to ensure good communication, follow-up of participants (OT, PT). Home booklets with simpler physical exercises and skills training demonstrated at informal meetings. Extra attention to new staff members to ensure treatment adherence  **Staff collaboration:** Goal assessment activity limitation (OT/PT), training in daily activities, adapt activity, environment modifications, exercise programs (home-care service)  **Dosage:** Average 10 wks (up to 3 mo)  **Adherence:** Use of home help 2.1 h/wk, most common profession assistant nurse 57.7%, RN 15%, OT 13.3%, PT 9.9% | Compensating help: assistance, safety alarm, meals on wheels, assistive technology (with OT (n=1), PT (n=5))  **Dosage:** Not time limited  **Adherence:** Use of home help 1.7 h/w, most common professions assistant nurse 67.2%, RN 24.2%, PT 2.6%, Social educator 1.5% |

Abbreviations: ADL=activities of daily living; BADL=basic ADL; CG=control group; d=days; F=number of female participants; h=hour(s); HRQL=health-related quality of life; IADL=instrumental ADL; IG=intervention group; M=mean; MD=median; mo=month(s); n=sample size or number of participants; OT=occupational therapist; PT=physiotherapist; RM=repetition maximum; RPE=rating perceived exertion; RPD rating perceived dyspnea; RN=registered nurse; wk(s)=week(s).
