# Supplementary material for: The components and effects of home rehabilitation on activities of daily living and physical performance of community dwelling older people with low physical performance – a systematic review and meta-analysis of randomized controlled trials
Source: BMC Geriatr. 2026 Jun 30;26:889. doi: 10.1186/s12877-026-07887-9 (PMC13321581; doi:10.1186/s12877-026-07887-9)
Supplement: Supplementary file 5 — Additional file 5. Outcome results of studies of Activity-based interventions. Outcomes each study. [file 12877_2026_7887_MOESM5_ESM.docx]

**Additional File 5.** Outcome results of studies of Activity-based interventions.

| **Study**  ***Intervention period*** | **Primary outcome(s)** | **Measurement** | **Outcome post-intervention** | | **Follow-up** | |
| --- | --- | --- | --- | --- | --- | --- |
| de Vriendt et al. ^47^  *8-10 weeks* | BADL  Reached power |  | **MD at 8-10 weeks** | **Between-groups difference in change (95% CI), *P* value at 8-10 weeks** |  |  |
|  |  | Mod WHO^‡^ (dependence) | IG 3.6  CG -3.1 | 6.7 (1.4 to 12.1) *P*=.013* | ------------ |  |
| Friedman et al. ^48^  *24 months* | No power calculation |  | **Regression: *B* Coefficient (SE) *z*/*t*, *P* value at 22 months** | | ------------ |  |
|  |  | BADL^†^ (dependence) | -0.25 (0.12), -2.07, *P*=.04* | | ------------ |  |
|  |  | IADL^†^ (dependence) | -0.16 (0.12), -1.33, *P*=.18 | | ------------ |  |
| Gitlin et al. ^28^  *6 months* | BADL, IADL, mobility)  Reached power |  | **M (SD) at 6 months** | **Between-group difference of adjusted means (95% CI), *P* value at 6 months** | **M (SD) at 12 months** | **Between-group difference of adjusted means (CI) *P* value at 12 months** |
|  |  | BADL^†^  (difficulty) | IG 1.58 (0.54)  CG 1.66 (0.63) | -0.13 (-0.24 to -0.01) *P*=.03* | Not reported | -0.10 (-0.21 to 0.02) *P*=.10 |
|  |  | IADL^†^  (difficulty) | IG 1.97 (0.69)  CG 2.07 (0.77) | -0.14 (-0.28 to 0.00) *P*=.04* | Not reported | -0.12 (-0.26 to 0.03) *P*=.13 |
|  |  | Mobility/transfer^†^  (difficulty) | IG 2.35 (0.72)  CG 2.41 (0.80) | -0.11^c^ *P*=.15 | Not reported | -0.14 (-0.29 to 0.01) *P*=.07 |
| Hagelskjaer et al.^29^  *2 months* | AMPS motor skills  Reached power |  | **LSmean (SE) at 10 weeks** | **Estimated treatment difference ΔLSmean (95% CI), *P* value at 10 weeks** | **LSmean (SE) at 26 weeks** | **Estimated treatment difference ΔLSmean (CI) *P* value at 26 weeks** |
|  |  | AMPS^‡^ motor skills | IG 0.3 (0.1)  CG 0.2 (0.1) | -0.1 (-0.3 to 0.1) *P*=.343 | IG 0.4 (0.1)  CG 0.1 (0.1) | -0.3 (-0.5 to -0.1) *P*=.018* |
|  |  | AMPS^‡^ process skills | IG 0.1 (0.1)  CG 0.1 (0.1) | -0.1 (-0.2 to 0.1) *P*=.404 | IG 0.1 (0.1)  CG 0.1 (0.1) | 0.0 (-0.2 to 0.1) *P*=.606 |
|  |  | ADL-I^‡^ performance  (B/IADL dependency) | IG 0.17 (0.08)  CG 0.01 (0.08) | -0.16 (-0.38 to 0.06) *P*=.164 | IG 0.19 (0.09)  CG 0.28 (0.09) | 0.09 (-0.15 to 0.34) *P*=.438 |
|  |  | ADL-I^‡^ satisfaction  (B/IADL dependency) | IG -0.10 (0.13)  CG -0.20 (0.13) | -0.10 (-0.46 to 0.27) *P*=.601 | IG 0.22 (0.14)  CG -0.09 (0.14) | -0.31 (-0.71 to 0.08) *P*=.120 |
| Nielsen et al.^30^  *3 months* | COPM performance  Reached power |  | **Change from baseline to 3 months M (SD)** | **Between-group differences from baseline to 3 months (95% CI), *P* value** | **Within-group difference from baseline to 6 months M (SD)** | **Between-group differences from baseline to 6 months (CI) *P* value** |
|  |  | COPM^‡^ performance | IG 1.87 (1.84)  CG 0.61 (1.94) | 1.26 (0.50 to 2.02) ***P***=**.001*** | IG 1.42 (1.91)  CG 0.44 (1.55) | 0.98 (0.27 to 1.70) *P*=.008* |
|  |  | COPM^‡^ satisfaction | IG 1.83 (2.06)  CG 1.12 (1.97) | 0.71 (-0.09 to 1.52) *P*=.08 | IG 2.08 (2.11)  CG 0.99 (1.82) | 1.09 (0.28 to 1.90) *P*=.009* |
|  |  | AMPS^‡^ motor skills | IG 0.33 (0.82)  CG 0.28 (0.74) | 0.05 (-0.30 to 0.39) *P*=.79 | IG 0.44 (0.68)  CG -0.00 (0.62) | 0.44 (0.14 to 0.74) *P*=.005* |
|  |  | AMPS^‡^ process skills | IG 0.17 (0.53)  CG 0.07 (0.51) | 0.11 (-0.13 to 0.34) *P*=.37 | IG 0.17 (0.49)  CG -0.02 (0.42) | 0.19 (-0.02 to 0.40) *P*=.08 |
| Sheffield et al.^31^  *3 months* | No power calculation |  | **Regression: Coefficient (SE) *t*, *P* value at 3 months** | |  |  |
|  |  | FIM^‡^  (dependency) | -2.14 (2.03), -1.05, *P*=.15 | | ------------ |  |
| Stark et al.^49^  *60 days* | No power calculation |  | **M (SD) at 6 months** | | **M (SD) at 12 months** | **Interaction effect (group x time) *F*, *P* value at 12 months** |
|  |  | I-HOPE^‡^ performance | IG 3.6 (0.7)  CG 3.1 (0.8) | | IG 3.6 (0.7)  CG 3.0 (0.9) | *F*=5.57, *P*=.005* |
|  |  | I-HOPE^‡^ satisfaction | IG 3.5 (0.9)  CG 3.0 (1.1) | | IG 3.4 (0.9)  CG 2.9 (1.1) | *F*=3.15, *P*=.046* |
|  |  | I-HOPE^†^  barrier | IG 16.8 (2.6)  CG 24.2 (2.7) | | IG 20.3 (2.9)  CG 28.0 (3.1) | *F*=4.13, *P*=.024* |
| Szanton et al.^33^  *6 months* | No power calculation |  | **M (SD) at 24 weeks** | **Effect size at 24 weeks** | ------------ |  |
|  |  | Katz ADL Index^†^  (difficulty) | IG 0.7 (0.8)  CG 2.1 (2.3) | 0.63 | ------------ |  |
|  |  | IADL^†^  (difficulty) | IG 1.2 (1.3)  CG 1.8 (1.9) | 0.62 | ------------ |  |
| Szanton et al.^34^  *5 months* | BADL, IADL  Reached power |  | **M (SE) at 5 months** | **Crude effect size RR (95% CI), *P* value / Adjusted effect size RR (CI) *P* value at 5 months** | **M (SE) at 12 months** | **Crude effect size RR (CI) *P* value / Adjusted effect size RR (CI) *P* value at 12 months** |
|  |  | Katz ADL Index^†^  (difficulty/ dependency) | IG 2.22 (0.26)  CG 2.83 (0.28) | 0.74 (0.57 to 0.97) *P*=.03* / 0.70 (0.54 to 0.93) *P*=.01* | IG 2.65 (0.30)  CG 2.67 (0.27) | 0.93 (0.72 to 1.21) *P*=.60 / 0.90 (0.68 to 1.18) *P*=.44 |
|  |  | IADL^†^  (difficulty/ dependency) | IG 3.86 (0.35)  CG 4.39 (0.34) | 0.90 (0.72 to 1.12) *P*=.35 /  0.83 (0.65 to 1.06) *P*=.13 | IG 4.50 (0.41)  CG 4.28 (0.37) | 1.06 (0.85 to 1.32) *P*=.61 / 1.03 (0.81 to 1.131) *P*=.80 |
| Szanton et al.^50^  *5 months* | BADL  Reached power |  | **Adjusted change**  **M (CI) at 5 months** | **Difference M (CI), (SE), *d*, *P* value at 5 months** |  |  |
|  |  | BADL^†^  (difficulty/ dependency) | IG -1.76 (-2.98 to -0.54)  CG -0.63 (-1.86 to 0.60) | -1.13 (-2.88 to 0.62), (0.89), -0.14 *P*=.204 | ------------ |  |
|  |  | IADL^†^  (difficulty/ dependency) | IG -0.93 (-1.60 to -0.26)  CG -0.58 (-1.26 to 0.10) | -0.35 (-1.31 to 0.61), (0.49), -0.08 *P*=.475 | ------------ |  |
|  |  | Mobility^†^  (difficulty/ dependency) | IG -0.53 (-0.84 to -0.21)  CG -0.02 (-0.34 to 0.30) | -0.51 (-0.97 to -0.05), (0.23), -0.25 *P*=.028* | ------------ |  |
| Whitehead et al.^36^  *8 weeks* | No power calculation |  |  | **Change from baseline IG-CG (SE) 95% CI adj. gender after 2 weeks** |  | **Change from baseline IG-CG (SE) CI adj. gender after 3 months / after 6 months** |
|  |  | Barthel Index^‡^ (dependence) | Not reported | 0.7 (1.08) -1.52 to 2.93 | Not reported | -0.13 (1.33) -2.91 to 2.65 /  0.28 (1.12) -2.06 to 2.61 |

†=A decrease in score indicates improvement. ‡=An increase in score indicates improvement. c) CI not reported. 95% CI. *P*<.05. *=significance favor IG.

Abbreviations: ADL-I=ADL Interview; AMPS=Assessment of Motor and Process Skills; BADL=basic activities of daily living; CG=control group; CI=confidence intervals; COPM=Canadian Occupational Performance Measure; FIM=Functional independence measure; IADL=instrumental activities of daily living; IG=intervention group; I-HOPE=In-Home Occupational Performance Evaluation; LSmean=Least squares mean; ΔLSmean=Least squares mean difference; M=mean; MD=mean difference; RR=risk ratio; SD=standard deviation; SE=standard error.
