# Supplementary material for: The components and effects of home rehabilitation on activities of daily living and physical performance of community dwelling older people with low physical performance – a systematic review and meta-analysis of randomized controlled trials
Source: BMC Geriatr. 2026 Jun 30;26:889. doi: 10.1186/s12877-026-07887-9 (PMC13321581; doi:10.1186/s12877-026-07887-9)
Supplement: Supplementary file 6 — Additional file 6. Outcome results of studies of Exercise-based interventions. Outcomes each study. [file 12877_2026_7887_MOESM6_ESM.docx]

**Additional File 6.** Outcome results of studies of Exercise-based interventions.

| **Study**  ***Intervention period*** | **Primary outcome(s)** | **Measurement** | **Outcome post-intervention** | | **Follow-up** | |
| --- | --- | --- | --- | --- | --- | --- |
| Bjerk et al.^51^  *12 weeks* | Secondary analyses |  | **Regression: Coefficient MD (SE) *P* value at 3 months** | **Additional improvement IG Coefficient MD (SE), *P* value at 3 months** |  |  |
|  |  | 30-s STS^†^ | 0.8 (0.4) p<.05* | 0.4 (0.6) | ------------ |  |
|  |  | 4MWT^‡^ | 0.06 (0.02) p<.01* | -0.00 (0.03) | ------------ |  |
|  |  | BBS^‡^ | 3.1 (0.8) p<.001* | 2.4 (1.2) p<.05* | ------------ |  |
| Boongird et al.^26^  *12 months* | No power calculation |  | **M at 3 months** | ***P* value at 3 months** | **M at 6 months** | ***P* value at 6 months** |
|  |  | 5STS^†^ | IG 18.8  CG 19.0 | *P*=.849 | IG 19.6  CG 18.7 | *P*=.254 |
|  |  | TUG^†^ | IG 17.1  CG 17.4 | *P*=.765 | IG 16.7  CG 16.8 | *P*=.785 |
|  |  | BBS^‡^ | IG 22.1  CG 22.0 | *P*=.828 | IG 21.6  CG 21.7 | *P*=.290 |
| Burton et al.^52^  *8-10 weeks* | Summary score#  Did not reach power |  | **M difference (SD) at 8 weeks** | ***z/t* (CI), *P* value at 8 weeks** |  |  |
|  |  | 5STS^†^ | IG -2.05 (5.00)  CG -0.33 (4.72) | -1.49 (-4.03 to 0.59), *P*=.142 | ------------ |  |
|  |  | Summary score^†^ | IG -0.55 (0.53)  CG -0.29 (0.68) | -1.76 (-0.56 to -0.03), *P*=.083 | ------------ |  |
| Cederbom et al.^27^  *10 weeks* | SPPB  Did not reach power |  | **M (SD) at 3 months** | **MD (95% CI),** *P* **value at 3 months** | **M (SD) at 6 months** | **MD (CI) *P* value at 6 months** |
|  |  | SPPB^‡^ | IG 5.9 (2.6)  CG 5.3 (3.1) | 0.1 (-0.47 to 0.71) p=.68 | IG 5.7 (2.9)  CG 5.2 (2.8) | 0.07 (-0.54 to 0.70) *P*=.81 |
| Clegg et al.^53^  *12 weeks* | No power calculation |  | **M (SD), change (SD) at 14 weeks** | **Unadjusted between-group MD (95% CI) /**  **Adjusted between-group MD (CI) at 14 weeks** |  |  |
|  |  | Barthel Index^‡^ (dependent) | IG 15.6 (4.0), -0.30 (2.4)  CG 15.0 (4.0), -0.8 (3.1) | 0.6 (-0.7 to 1.8) /  0.6 (-0.7 to 1.8) | ------------ |  |
|  |  | TUG^†^ | IG 62.4 (77.7), -10.4 (64.0)  CG 97.0 (116.7), -39.1 (90.6) | 28.7 (-8.2 to 65.5) /  28.6 (-8.5 to 65.9) | ------------ |  |
| Courtney et al.^54^  *24 weeks* | No power calculation |  | **M (SD) at 4 weeks** |  | **M (SD) at 12 weeks /**  **M (SD) at 24 weeks** | **Repeated-measures over time between groups *F*, *P* value at 24 weeks** |
|  |  | Katz ADL Index^†^ (dependence) | IG 0.07 (0.25)  CG 0.69 (1.21) |  | IG 0.18 (0.68)  CG 0.75 (1.25) /  IG 0.16 (0.42)  CG 1.27 (1.71) | *F*=9.733, *P*<.001* |
|  |  | IADL^†^ (dependence) | IG 1.47 (1.50)  CG 3.29 (2.03) |  | IG 1.27 (1.67)  CG 3.56 (1.98) /  IG 1.13 (1.71)  CG 4.33 (2.15) | *F*=30.645, *P*<.001* |
|  |  | WIQ distance^‡^ | IG 53.62 (25.88)  CG 29.90 (30.21) |  | IG 54.83 (27.79)  CG 21.59 (26.17) /  IG 62.89 (28.17)  CG 19.93 (24.93) | *F*=19.49, *P*<.001* |
|  |  | WIQ speed^‡^ | IG 41.30 (21.90)  CG 22.09 (22.52) |  | IG 44.62 (24.57)  CG 17.89 (20.67) /  IG 48.56 (25.75)  CG 16.58 (19.94 | *F*=17.66, *P*<.001* |
|  |  | WIQ stairs^‡^ | IG 46.73 (29.35)  CG 26.06 (25.65) |  | IG 51.23 (28.13)  CG 24.40 (22.28)/  IG 57.20 (27.80)  CG 22.18 (24.32) | *F*=16.98, *P*<.001***** |
| Garbin et al.^55^  *60 days* | SPPB  Reached power |  | **LSmean Change at 60-day (CI)** | **Estimated difference in change (95% CI), *P* value at 60 days** |  |  |
|  |  | SPPB^‡^ | IG 1.53 (1.00 to 2.05)  CG 1.39 (0.89 to 1.88) | -0.14 (-0.82 to 0.54) *P*=.68 | ------------ |  |
|  |  | mPPT^‡^ | IG 3.39 (2.12 to 4.66)  CG 3.82 (2.58 to 5.05) | 0.42 (-1.23 to 2.08) *P*=.61 | ------------ |  |
|  |  | Step count^‡^ | IG 758.74 (-861.62 to 2379.09  CG 443.69 (-465.98 to 1353.36) | 314.19 (-46.78 to 675.16) *P*=.39 | ------------ |  |
|  |  | Gait speed^‡^ | IG 0.13 (0.09 to 0.17)  CG 0.14 (0.11 to 0.18) | 0.01 (-0.04 to 0.06) *P*=.63 | ------------ |  |
|  |  | Gait speed fast^‡^ | IG 0.20 (0.13 to 0.26)  CG 0.17 (0.11 to 0.23) | -0.03 (-0.11 to 0.05) *P*=.47 | ------------ |  |
| Sherrington et al.^56^  *12 months* | Summary Performance score  Did not reach power |  | **M (SD) at 12 months** | **M difference between groups (CI), *P* value at 12 months** |  |  |
|  |  | Ease of personal care tasks^‡^ | IG 40.5 (8.6)  CG 38.7 (10.3) | 1.5 (-0.26 to 3.33), *P*=.094 | ------------ |  |
|  |  | SPPB^‡^ | IG 7.41 (2.02)  CG 6.89 (1.92) | 0.50 (0.11 to 0.90), *P*=.013* | ------------ |  |
|  |  | Summary Performance score^‡^ | IG 2.06 (0.52)  CG 1.94 (0.56) | 0.13 (0.04 to 0.21), *P*=.004* | ------------ |  |
| Siemonsma et al.^57^  *3 months* | B/IADL GARS  Reached power |  |  | **Difference M change between-group (95% CI) (FTE and PPT) at 12 months** |  |  |
|  |  | GARS^†^ (B/IADL dependence) | Not reported | -0.4 (-2.3 to 3.0) *P*=.795 | ------------ |  |
|  |  | Katz-15^†^ (B/IADL dependence) | Not reported | -0.4 (-1.1 to 0.4) *P*=.339 | ------------ |  |
| Stevens-Lapsley et al.^58^  *1 month* | No power calculation |  | **M (CI) change at 30 days** | **Between-group difference M (95% CI) *P* value at 30 days** | **M (CI) change at 60 days** | **Between-group difference M (CI) *P* value at 60 days** |
|  |  | SPPB^‡^ | IG 1.87 (0.52 to 3.22)  CG 0.96 (-0.52 to 2.44) | -0.91 (-2.93 to 1.10) *P*=.36 | IG 2.94 (1.46 to 4.42)  CG 0.38 (−1.06 to 1.83) | -2.56 (-4.65 to -0.46) *P*=.02* |
|  |  | mPPT^‡^ | IG 5.54 (2.85 to 8.23)  CG 0.66 (−2.29 to 3.60) | -4.88 (-8.87 to -0.89) *P*=.02* | IG 6.18 (3.04 to 9.32)  CG 0.98 (−2.12 to 4.09 | -5.20 (-9.62 to -0.77) *P*=.02* |
|  |  | 6-minute walk test (m)^†^ | IG 62.46 (15.19 to 109.73)  CG 19.88 (−43.57 to 83.33) | -42.58 (-125.11 to 39.94) *P*=.29 | IG 119.65 (52.55 to 186.76)  CG 19.28 (−59.67 to 98.22 | -100.38 (-208.10 to 7.34) *P*=.07 |
|  |  | Walking speed^†^ | IG 0.23 (0.11 to 0.34)  CG 0.10 (-0.03 to 0.22) | -0.13 (-0.30 to 0.04) *P*=.12 | IG 0.36 (0.21, 0.51)  CG 0.14 (−0.02, 0.29) | - 0.22 (-0.44 to -0.01) *P*=.04* |
| Stevens-Lapsley et al.^32^  *30 days* | Secondary analyses |  | **M (SD) at 30 days** | ***P* value at 30 days** | **LSmean Change at 60-day (CI)** | **Estimated difference in change (SE) *P* value at 60 days** |
|  |  | SPPB^‡^ | Not reported | Not reported | IG 0.83 (0.22-1.44)  CG 1.04 (0.42-1.65) | -0.20 (0.44) *P*=.64 |
|  |  | mPPT^‡^ | Not reported | Not reported | IG 2.72 (1.50-3.93)  CG 2.78 (1.57-4.00) | -0.07 (0.87) *P*=.94 |
|  |  | TUG^†^ | Not reported | Not reported | IG -3.03 (-5.68 to -0.38)  CG -3.20 (-5.85 to -0.55) | 0.17 (1.9) *P*=.93 |
|  |  | Step counts^‡^ | Not reported | Not reported | IG 628 (169, 1088)  CG 309 (-184, 802) | 319 (CI 95% -368, 1006) *P*=.36 |
| Whitney et al.^59^  *56 days* | No power calculation |  | **M change (SD) at 56 days** | **Between-group difference effect size, *Z*, *P* value at 56 days** |  |  |
|  |  | ADL^†^ (B/IADL dependent) | IG 10.0 (3.7)  CG 7.9 (4.1) | 0.25, *Z*=2.26, *P*=.24 | ------------ |  |
| Yang et al.^37^  *6 months* | Secondary analyses |  | **M (SD) at 6 months** | **MD between groups (95% CI) *P* value at 6 months** |  |  |
|  |  | 5STS^†^ | IG 9.76 (2.37)  CG 10.93 (3.54) | -1.08 (-1.83 to -0.33) *P*=.01* | ------------ |  |
|  |  | FRT^‡^ | IG 28.39 (4.86)  CG 26.87 (4.30) | 2.95 (1.75 to 4.15) *P*<.001* | ------------ |  |
|  |  | Step Test^‡^ | IG 15.54 (3.99)  CG 14.41 (3.77) | 2.10 (1.17 to 3.02) *P*<.001* | ------------ |  |
|  |  | 6MWT^‡^ | IG 61.39 (13.33)  CG 62.15 (13.91) | 2.07 (-1.49 to 5.63) *P*=.25 | ------------ |  |

†=Decrease in score indicates improvement. ‡=Increase in score indicates improvement. #=Summary score (Functional reach test, Sit-to-stand test, Timed up-and-go test, Tandem walk). 95% CI. *P*<.05. *=significance favor IG.

Abbreviations: 4MWT=4-meter walk test (m/sec); 5STS=Five-times sit-to-stand test; 6MWT=6-meter walk test (m/min); 30-s STS=30-seconds sit-to-stand test; ADL=activities of daily living; BADL=basic ADL; BBS=Berg balance score; CG=control group; CI=confidence intervals; FRT=Functional reach test; GARS Groningen Activities Restriction; IADL=instrumental ADL; IG=intervention group; M=mean; MD=mean difference; mPPT=modified physical performance test; SD=standard deviation; SE=standard error; SPPB=Short physical performance battery; TUG=Timed up-and-go test; WIQ=Walking Impairment Questionnaire.
