# Supplementary material for: The components and effects of home rehabilitation on activities of daily living and physical performance of community dwelling older people with low physical performance – a systematic review and meta-analysis of randomized controlled trials
Source: BMC Geriatr. 2026 Jun 30;26:889. doi: 10.1186/s12877-026-07887-9 (PMC13321581; doi:10.1186/s12877-026-07887-9)
Supplement: Supplementary file 7 — Additional file 7. Outcome results of studies of Reablement-based interventions. Outcomes each study. [file 12877_2026_7887_MOESM7_ESM.docx]

**Additional File 7.** Outcome results of studies of Reablement-based interventions.

| **Study**  ***Intervention period*** | **Primary outcome(s)** | **Measurement** | **Outcome during intervention** |  | **Follow-up** | |
| --- | --- | --- | --- | --- | --- | --- |
| Gustafsson et al. ^60^  *3 months* | Secondary analyses |  | **M change from baseline 6 months** | ***P* value at 6 months** |  | |
|  |  | COPM^‡^ performance | IG 6.80  CG 6.16 | *P*=.60 | ------------ | |
|  |  | COPM^‡^ satisfaction | IG 6.85  CG 6.45 | *P*=.28 | ------------ | |
|  |  | SPPB‡ | IG 4.41  CG 4.89 | *P*=.38 | ------------ | |
| Rooijackers et al. ^61^  *Not reported* | No power calculation |  | **M (CI) at 12 months** | **Adjusted MD (95% CI),** *P* **value at 12 months** |  |  |
|  |  | GARS^†^ (B/IADL dependence) | IG 40.9 (38.6 to 43.2)  CG 42.8 (40.5 to 45.0) | -1.1 (-2.9 to 0.8), *P*=.252 | ------------ | |
|  |  | GARS^†^ (BADL dependence) | IG 20.8 (19.4 to 22.1)  CG 21.7 (20.4 to 23.0) | -0.6 (-1.7 to 0.5), *P*=.267 | ------------ | |
|  |  | GARS^†^ (IADL dependence) | 19.0 (17.9 to 20.1) 20.0 (18.9 to 21.0) | -0.4 (-1.4 to 0.6), *P*=.406 | ------------ | |
|  |  | SPPB^‡^ | IG 3.9 (3.4 to 4.5)  CG 4.4 (4.0 to 4.9) | -0.6 (-1.1 to -0.1), *P*=.028* | ------------ | |
| Tuntland et al. ^35^  *Up to 3 months* | COPM  Reached power |  | **M (CI)**  **After 3 months** | **Adjusted effect size, Treatment effect MD (95% CI), *P* value**  **After 3 months** | **M (CI)**  **After 9 months** | **Adjusted effect size, Treatment effect MD, *P* value /**  **Overall treatment effect MD (CI), *P* value**  **After 9 months** |
|  |  | COPM^‡^ performance | IG 6.9 (6.1 to 7.8)  CG 5.5 (4.7 to 6.3) | 0.8, 1.5 (0.3 to 2.8) *P*=.02* | IG 6.3 (5.0-7.6)  CG 4.8 (4.1-5.5) | 0.7, 1.4 (0.2 to 2.7) *P*=.03* /  1.5 (0.4 to 2.6) *P*=.01* |
|  |  | COPM^‡^ satisfaction | IG 6.7 (5.9 to 7.6)  CG 6.0 (5.3 to 6.8) | 0.7, 1.0 (-0.3 to 2.2) *P*=.13 | IG 6.5 (5.2-7.8)  CG 5.2 (4.5-5.9) | 0.9, 1.4 (0.1 to 2.7) *P*=.03* /  1.2 (0.1 to 2.3) *P*=.04* |
|  |  | TUG^†^ | IG 19.6 (14.2 to 25.1)  CG 17.9 (14.0 to 21.8) | 0.1, -0.4 (-4.3 to 3.5) *P*=.82 | IG 19.9 (14.7–25.0)  CG 18.1 (13.4–22.8) | 0.1, 0.3 (-3.7 to 4.3) ***P***=.88 /  -0.1 (-3.8 to 3.5) ***P***=.96 |

†=Decrease in score indicates improvement. ‡=Increase in score indicates improvement. 95% CI. p<.05. *=significance favor IG.

Abbreviations: BADL=basic activities of daily living; CG=control group; CI=confidence intervals; COPM=Canadian Occupational Performance Measure; IADL=instrumental ADL; IG=intervention group; M=mean; MD=mean difference; GARS Groningen Activities Restriction; SPPB=Short physical performance battery; TUG=Timed up-and-go test.
