# Supplementary material for: The components and effects of home rehabilitation on activities of daily living and physical performance of community dwelling older people with low physical performance – a systematic review and meta-analysis of randomized controlled trials
Source: BMC Geriatr. 2026 Jun 30;26:889. doi: 10.1186/s12877-026-07887-9 (PMC13321581; doi:10.1186/s12877-026-07887-9)
Supplement: Supplementary file 9 — Additional file 9. Funnel plot Exercise-based interventions. [file 12877_2026_7887_MOESM9_ESM.pdf]

Additional file 9. Funnel Plots Exercise-based interventions

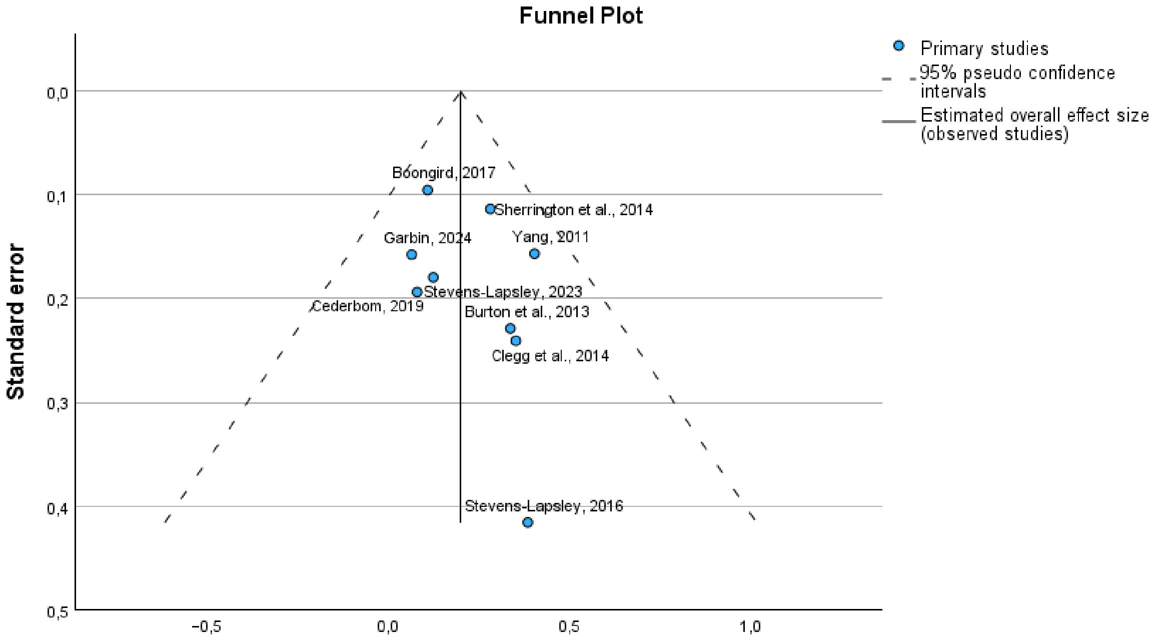

Additional file 9. Funnel Plot meta-analysis of Exercise-based intervention effects on physical performance.
